# Structural Evidence for Inter-Residue Hydrogen Bonding Observed for Cellobiose in Aqueous Solution

**DOI:** 10.1371/journal.pone.0045311

**Published:** 2012-10-02

**Authors:** William B. O'Dell, David C. Baker, Sylvia E. McLain

**Affiliations:** 1 Department of Chemistry, University of Tennessee, Knoxville, Tennessee, United States of America; 2 Department of Biochemistry, University of Oxford, Oxford, United Kingdom; National Cancer Institute at Frederick, United States of America

## Abstract

The structure of the disaccharide cellulose subunit cellobiose (4-*O*-β-D-glucopyranosyl-D-glucose) *in solution* has been determined via neutron diffraction with isotopic substitution (NDIS), computer modeling and nuclear magnetic resonance (NMR) spectroscopic studies. This study shows direct evidence for an intramolecular hydrogen bond between the reducing ring HO3 hydroxyl group and the non-reducing ring oxygen (O5′) that has been previously predicted by computation and NMR analysis. Moreover, this work shows that hydrogen bonding to the non-reducing ring O5′ oxygen is shared between water and the HO3 hydroxyl group with an average of 50% occupancy by each hydrogen-bond donor. The glycosidic torsion angles *φ_H_* and *ψ_H_* from the neutron diffraction-based model show a fairly tight distribution of angles around approximately 22^°^ and −40^°^, respectively, in solution, consistent with the NMR measurements. Similarly, the hydroxymethyl torsional angles for both reducing and non-reducing rings are broadly consistent with the NMR measurements in this study, as well as with those from previous measurements for cellobiose in solution.

## Introduction

Conversion of plant cellulose into ethanol has been industrially achievable since the late Nineteenth Century. However, the near-insolubility of cellulose in aqueous solvents initially required physical separation of pure cellulose from plant material and harsh acid hydrolysis to produce the glucose used in fermentation.[1] Today, interest lies in using complete lignocellulosic biomass as an ethanol feedstock, but the challenge of cellulosic recalcitrance to aqueous solvation and requirements for extensive physical and/or chemical pretreatment of the biomass remains. This is despite the active study of cellulose's chemical structure which dates back to the beginnings of modern molecular structure analysis.[2]–[4] While great progress has been made in understanding cellulose in the solid state,[5]–[7] cellulosic recalcitrance largely prevents structural studies of cellulose–water interactions. An example of the difficulty of studying cellulose in aqueous environments comes from the field of NMR spectroscopy. Using solution-state NMR experiments it is possible to determine protein structures with accuracies matching that of crystallography, but only cello-oligomers in the range of 2–6 glucose subunits have been extensively characterized in water solvent due to poor solubility.[8] Small-angle neutron and X-ray scattering experiments have revealed the bulk morphology of cellulose fibers with varying degrees of hydration,[9], [10] and hydrogen bonding in and among cellulose chains in dry and aqueous suspensions of microcrystals,[11]–[13] but a structural description of cellulose–water or cello-oligomer–water interactions on the atomic length scale (1–10 Å) in solution has yet to be attained.

Unlike higher cello-oligosaccharides, cellobiose with two glucose subunits exhibits considerable solubility in water, making it an ideal model molecule for investigations in solution. Cellobiose and methyl cellobioside in aqueous solutions have been studied extensively by NMR spectroscopy where various measures of coupling constants[14]–[19] have been determined. More recent studies have sought to quantify the populations of hydrogen bonds across the β-(1→4) linkages in solutions of disaccharides.[18]–[21] Cellobiose has also been investigated extensively by computation, from early stereochemical approaches[22] and molecular dynamics (MD) simulations[23]–[25] to modern quantum mechanical methods.[26], [27] More recently cellobiose analogues have been studied by spectroscopic methods combined with Car–Parrinello-type simulations.[28]

**Figure 1:**
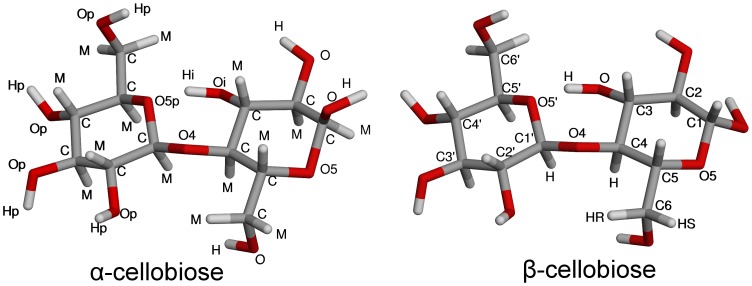
Molecular structure of α- and β-cellobiose. α-Cellobiose is labeled with atom types from the EPSR simulation, and β-cellobiose is labeled according to the IUPAC recommended nomenclature.

Despite these extensive studies, there is still debate about the structure of cellobiose in solution–specifically with respect to its conformation about the glycosidic linkage as well as its hydrogen-bonding structure. There is a particular question concerning whether or not an internal hydrogen bond between the non-reducing ring oxygen (O5′) and the adjacent reducing ring hydroxyl group (HO3; see Fig. 1)[17]–[19], [25]–[27], [29], [30] is present in cellobiose or methyl cellobioside and if the persistence of this intramolecular hydrogen bond contributes to the low solubility of cellulose in water. Part of the reason for this continuing uncertainty in the solution structure of cellobiose is that hydrogen bonding in solution is difficult to measure by most experimental techniques.

In this work, NDIS augmented by computer simulation has been used in combination with NMR spectroscopy to determine the structure of cellobiose in aqueous solution. NDIS can measure the hydrogen bonding of molecules in aqueous solutions on the atomic-length scale and has been one of the premier techniques for structural determinations of hydrogen-containing liquids due to the ‘sensitivity’ of the neutron to hydrogen and deuterium. NDIS also has the advantage of being a direct structural technique, analogous to crystallography, which does not rely on structural interpretation of dynamical data as is the case with spectroscopy. By combining neutron scattering and computation with NMR spectroscopy, which provides an assessment of the cellobiose conformations, a rigorous structural assessment of cellobiose hydrogen bonding in water can be realized.

## Theory

### NDIS

NDIS has been used to investigate the structure of hydrogen-containing liquids such as anhydrous HF,[31] water[32] and organic solvents[33]–[36] as well as aqueous solutions of polar and ionic species[37]–[41] including several biological molecules.[42]–[49] Unlike X-rays where the scattering intensity is proportional to atomic size, neutrons are scattered by virtue of a neutron–nucleus interaction. This interaction is independent of atomic size and is of the same order of magnitude for both light and heavy atoms. As such, hydrogen atoms scatter neutrons with a relatively large intensity compared to hydrogen scattering from X-rays. Furthermore, because of the nuclear dependence, neutron scattering intensities vary for isotopes of the same element.[50] In the case of hydrogen the scattering intensity difference between hydrogen and deuterium is relatively large and can be exploited by measuring a series of isotopically *unique* yet chemically *equivalent* samples yielding multiple distinct measurements of the same chemical system.

The quantity measured in a neutron diffraction experiment is the total scattering structure factor, *F*(*Q*),
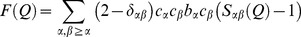
(1)where *c_i_* and *b_i_* are the relative concentration and scattering length of atom *i*,[50] respectively; *δ_αβ_* is the Kroneker delta function introduced to avoid double counting of like-atom pairs, and *S_αβ_*(*Q*) is the partial structure factor for the *α*–*β* atom pair. *Q* is the change in magnitude of the incident wave vector of the neutrons when they are scattered from the sample where *Q* = 4*π*sin(*θ*)/*λ* with 2*θ* being the scattering angle relative to the incident neutron beam and *λ* the incident neutron wavelength.

Eq. 1 describes the sum of all the partial structure factors, for which there are *m*(*m*+1)/2 for *m* number of structurally unique atoms in the system. The Fourier transform of *S_αβ_*(*Q*) gives the distribution of atomic separations (distances) in real space on an atomic scale (Å) as a radial distribution function (RDF)
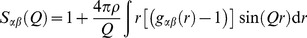
(2)where *ρ* is the atomic number density of the sample and *g_αβ_*(*r*) is the RDF for the *α*–*β* atom pair.

To characterize the average local structure of a liquid, the RDF for an atom pair can be integrated yielding the average coordination number of atoms *β* around atoms *α*, 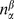, over the distance range *r_1_* to *r_2_*, namely
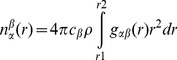
(3)


The coordination number is usually calculated from atom *α* at the origin (*r_1_* = 0) to the distance of the first minimum (*r_2_*) after the first obvious maximum in the RDF.

### Empirical Potential Structure Refinement (EPSR)

With NDIS it is only possible to extract all of the RDFs experimentally from systems with a small number of unique atoms such as HF[31] or water.[32] In practice, it is not feasible to measure a complete set of chemically equivalent yet isotopically unique samples due to (a) the limited availability of isotopes with significantly different neutron scattering lengths and (b) the difficulty of selectively labeling each unique atom in the system. In order to obtain a full set of RDFs from complex systems, a model-based approach known as EPSR can be used to augment the NDIS experiment by calculating a full set of RDFs that are consistent with the measured diffraction data.[51]

The EPSR method begins with a standard Monte Carlo simulation of the sample structure based on a set of reference potentials. EPSR perturbs these initial reference potentials thus creating new potentials whose magnitudes are proportional to the difference between the measured total structure factors and those calculated from the model. EPSR then uses these new *empirical* potentials in the Monte Carlo simulation. This refinement process proceeds iteratively and results in a model that is consistent with a set of measured diffraction data. It should be noted that, while EPSR provides a model consistent with the data, it is not necessarily a definitive model of the systems in question. As is true with any modeling technique which determines the structure present in a liquid, as much knowledge as possible about the solution must be introduced into the EPSR model such as charges, molecular structures and overlap restrictions (as is done in the present case), in order to provide a physically realistic model of the physical structure. RDFs can be calculated for each unique atom pair from the resulting model. Additionally, spherical harmonic expansion of the calculated RDFs can be performed to generate spatial density functions (SDFs) that describe the location of molecules or portions of molecules relative to one another in three dimensions. A more detailed description of EPSR is given elsewhere,[51] and further details relevant to the derivation of SDFs (see below) are provided in the Supporting Information File S1.

## Materials and Methods

### NDIS Experiments

Neutron diffraction measurements were performed using the Small Angle Neutron Diffractometer for Amorphous and Liquid Samples (SANDALS) located at the ISIS facility (Rutherford Appleton Laboratory, STFC, UK) on an isotopomeric series of cellobiose–water solutions each at a molecular ratio of 1∶63 cellobiose:water (∼0.88 M) at standard ambient temperature and pressure (298±0.1 K, 1 bar). Isotopic H/D substitution was performed on the cellobiose hydroxyl groups and on the water solvent. Samples were prepared from cellobiose (β-d-glucopyranosyl-(1→4)-d-glucopyranose, 99%, Sigma–Aldrich) and ultra-pure water (Milipore) or from a sample of cellobiose previously lyophilized from deuterium oxide (D_2_O) (99.9% D, Cambridge Isotopes) and fresh D_2_O (99.9%, Sigma–Aldrich). Seven isotopically unique cellobiose–water solutions were studied; the isotopic composition of each sample is listed in the Supporting Information File S1.

The solutions were prepared by weight and transferred to sample cans constructed from a Ti/Zr alloy with a flat plate geometry and 1 mm sample thickness. Ti/Zr cans give very little scattering, thus simplifying data analysis, due to cancellation from the positive and negative coherent neutron scattering lengths of zirconium and titanium, respectively.[52] Data acquisition for each sample was conducted for ∼1500 µA proton current (8–10 h) to give adequate statistics for the total structure factors. Raw data were obtained for the samples, empty containers, instrument background, and a vanadium standard in order to ensure accurate background subtraction and normalization. SANDALS is also equipped with a neutron transmission monitor that measures the neutron cross-sectional area of samples relative to the incident beam allowing for an absolute measure of scattering from the sample. The scattering observed for each isotopically labeled solution was within 10% of the expected theoretical level. Data were converted to *F*(*Q*) after appropriate corrections for neutron absorption, multiple scattering, and inelasticity effects using the program GUDRUN, which is based on the ATLAS suite of programs available at the ISIS facility.[53]

### NMR Spectroscopy

NMR studies were conducted to probe the molecular conformation of cellobiose in aqueous solution. One-dimensional (1D) ^1^H NMR spectroscopy and two-dimensional (2D) NMR correlation spectroscopy were performed using a Varian Inova spectrometer operating at 600 MHz for ^1^H and 150 MHz for ^13^C. The instrument was equipped with a triple-channel cryoprobe and *z*-axis gradients. 1D ^13^C NMR spectroscopy was performed using a Bruker AMX spectrometer operating at 100 MHz for ^13^C and equipped with a broadband X-channel inverse geometry probe. The sample temperature was regulated at 298±0.5 K for all measurements. ^1^H and ^13^C resonances were interpreted according to Roslund *et al*.[54] Offline spectral processing, measurement of coupling constants and 1D and 2D resonance integration were performed using MestReC 4.9 (MestReLab Research, SL).

Cellobiose exists in two anomeric forms in aqueous solution at an equilibrium ratio of 38∶62 α-:β-cellobiose (Fig. 1).[54] Possible shifts in this equilibrium due to concentrations or isotopic substitutions similar to those used in the NDIS experiments were investigated by 1D ^13^C spectroscopy. Samples of cellobiose lyophilized from D_2_O were prepared in fresh D_2_O at concentrations of 15 mM and 0.88 M. These two concentrations were chosen to represent a “typical” NMR measurement (15 mM) and the concentration measured by NDIS (0.88 M), thus allowing a direct comparison of results from the two methods. ^13^C NMR spectra were acquired using inverse-gated ^1^H decoupling over six hours (∼8,000 transients) to obtain signal-to-noise ratios exceeding 30∶1, thus allowing for accurate integration of the signals arising from the anomeric carbons. Anomeric ratios of ∼40∶60 α-:β-cellobiose were observed at both concentrations.

**Figure 2:**
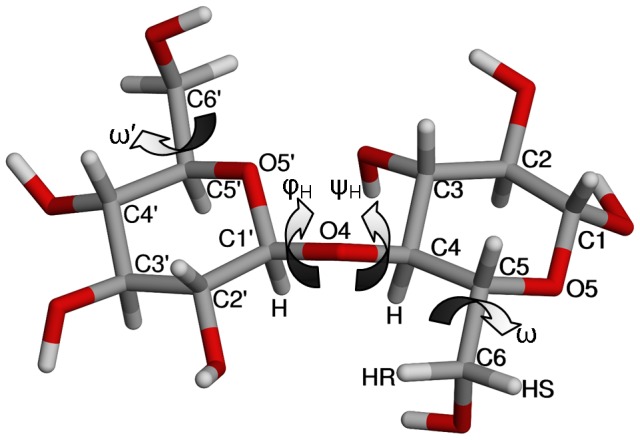
Definitions of the torsion angles *φ_H_*, *ψ_H_*, *ω* and *ω′*. Angles *φ_H_* and *ψ_H_* are the glycosidic torsional angles and *ω* and *ω′* are the hydroxymethyl torsional angles

J-coupling-modulated ^1^H, ^13^C gHMBC (J-mod gHMBC) experiments were performed to determine the magnitudes of the interglycosidic coupling constants *^3^J_H1’,C4_* and *^3^J_H4,C1'_*. The J-mod gHMBC experiment,[55] yields a 2D spectrum showing heteronuclear correlations through two or more bonds, and the absolute intensity of each correlation cross-peak is proportional to sin(*π^n^J_H,X_τ*) where *τ* represents a variable mixing time for polarization transfer. Performing a series of J-mod gHMBC experiments with varied mixing times and integrating the 1D projections of the cross-peaks of interest from each spectrum produces a set of intensity values as a function of *τ* that can be fit by non-linear regression to extract the value of *^n^J_H,X_*. J-mod gHMBC spectra of 15 mM and 0.88 M cellobisoe in D_2_O were recorded for *τ* values ranging from 20–200 ms. Coupling constants derived by non-linear regression were related to the torsion angles *φ_H_* (H1′–C1′–O4–C4) and *ψ_H_* (C1′–O4–C4–H4), illustrated in Fig. 2, using the Karplus-type relationship for H–C–O–C dihedral angles previously derived (Eq 4).[56]

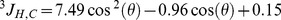
(4)


Determinations of the cellobiose hydroxymethyl (CH_2_OH) rotamer populations were made using the method reported by Serianni and co-workers[57] for measured values of *^3^J_H5,H6R_* and *^3^J_H5,H6S_*. This method assumes that the measured coupling constants are weighted averages resulting from the population of three preferred conformations of the ω dihedral angle (O5–C5–C6–O6 = +65°, −65° or ±180°). The following Karplus-type relationships for *^3^J_H5,H6R_* and *^3^J_H5,H6S_* are solved for each of the preferred conformations, and the calculated coupling constants are used to factor out the contributions of the preferred conformations to the measured, average coupling constant (Eq. 's 5 and 6).
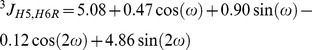
(5)

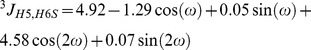
(6)


### EPSR

An EPSR modeling box was constructed using 20 cellobiose molecules (8 α-cellobiose and 12 β-cellobiose) and 1260 water molecules for a 1∶63 cellobiose:water molecular ratio at a density of 0.103018 atoms Å^−3^. Initial atomic coordinates, bond distances, and angles for the cellobiose molecules were taken from the β-cellobiose crystal structure[58] with the anomeric configuration inverted at C1 for α-cellobiose molecules and all O–H bond distances increased to 1.0 Å. Several non-bonding distance constraints were added to the cellobiose molecules in order to reproduce the energetically preferred ^4^ *C*_1_ chair conformation of the hexopyranose rings.[59]–[61] These additional constraints were introduced through specifying values for backbone torsion angles and creating non-bonding energy potentials between atoms one and four of each torsion. The constraints were necessary since the relatively weak neutron scattering intensity from carbon atoms[50] compared with hydrogen or deuterium and identical isotopic labeling of numerous atomic sites in the experiment reduced the conformational information available to guide the EPSR simulation process. It should be noted that these constraints did not include any X–X–O–H torsions that would influence hydroxyl group orientation nor did they constrain the intramolecular (or inter-residue) flexibility in the cellobiose molecules. The constraints are listed in full in the Supporting Information File S1. Constraints specific to the glycosidic linkage were also added to guide the EPSR model with values derived from the J-mod gHMBC experiments (See foregoing Experimental section, NMR spectroscopy).[55] Finally, a non-bonding distance constraint of 2.205 Å between the H1' and H4 atoms (Fig. 1) was also introduced to further stabilize the conformation of the glycosidic linkage.

The EPSR reference potentials used were Single Point Charge/Extended (SPC/E) model for water molecules (Ow and Hw),[62] and Lennard-Jones potentials and atomic charges from a modified CHARMM force-field for cellobiose molecules.[60] Values for parameters of the potentials are listed in the Supporting Information File S1. The atomic labels for cellobiose in the EPSR model are shown in Fig. 1 along with the IUPAC-recommended nomenclature for cellobiose atoms, which is used herein except where explicitly stated otherwise.[63] The simulation was conducted until ∼50,000 unique configurations of the minimized structural model were accumulated. The corrected neutron diffraction data, the EPSR fitted total structure factors, and the residuals between the data and fit are shown in the Supporting Information File S1.

## Results and Discussion

### Cellobiose Conformation in the EPSR Model

**Figure 3:**
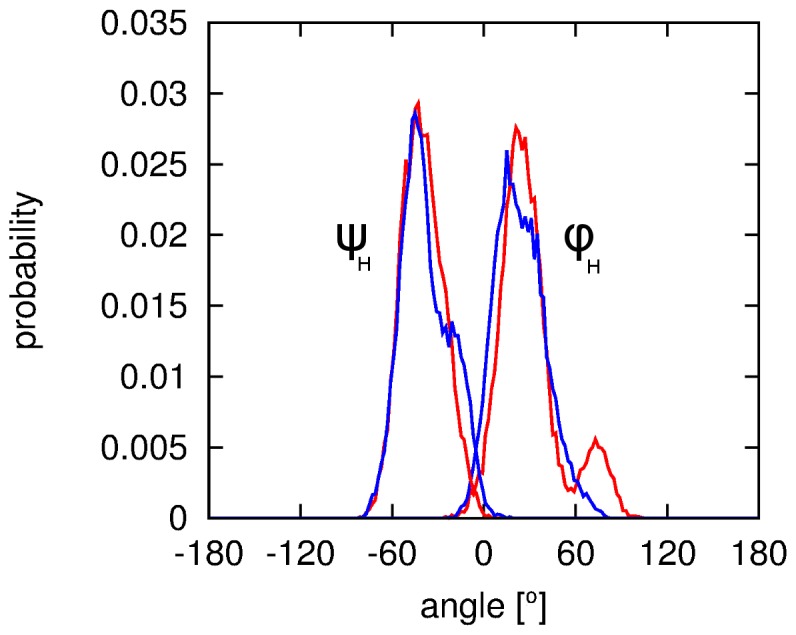
Distribution of glycosidic (*φ_H_*, *ψ_H_*) torsion angles from cellobiose in the EPSR model of the neutron diffraction data. Torsions from α-cellobiose are shown in red while those from β-cellobiose are shown in blue.

Flexibility in cellobiose primarily arises from rotations about the β-(1→4) glycosidic linkage and the orientation of the exocyclic hydroxymethyl groups about the C5(')–C6(') bonds. The torsion angles related to these flexibilities, *φ_H_ ψ_H_* and *ω(')*, are illustrated in Fig. 2. Constraints were imposed in the EPSR model as described above to keep the glycosidic conformation similar to that observed by NMR measurements. However, despite the constraints, these angles remained flexible in the model in order to accurately reproduce the NDIS data. Table 1 compares values of *φ_H_* and *ψ_H_* obtained via crystallography,[58] previous NMR mesurements,[16], [64] and density functional theory (DFT) studies[27] with the average values from the NMR measurements in this study and the EPSR model of the measured NDIS data. The EPSR average has been performed in two ways: (a) by taking the average of the two peak heights as seen in Fig. 3, and (b) by fitting these peaks to a Gaussian function in order to obtain a peak maximum with the results of these methods reported in Table 1.

**Table 1:** Comparison of glycosidic torsion angles *φ_H_* and *ψ_H_* from NMR studies and the EPSR model.

Molecule(s)	Method	^3^ *J*_C4,H1_(Hz)	*^3^J_C1',H4_*(Hz)	*φ_H_*(°)	*ψ_H_*(°)
β-cellobiose	X-ray crystallography[58]	–	–	42.3	−17.9
α/β-cellobiose	NMR[63]	3.80	4.60	40.1	−44.8
α/β-cellobiose	NMR[16]	4.08	4.75	37.7	−43.8
α/β-cellobiose	DFT[26] ^b^	–	–	20	−20
α/β-cellobiose (15 mM)	NMR^a^	4.30±0.72	4.44±2.24	35.8±6.4	−45.9±17.3
α/β-cellobiose (0.88 M)	NMR^a^	4.58±0.16	5.32±1.15	33.3±1.5	−39.7±8.5
α-cellobiose (0.88 M)	EPSR (average)^a^	–	–	29.9	−40.0
	EPSR (Gaussian fit)^a^			24.1	−41.1
β-cellobiose (0.88 M)	EPSR (average)^a^	–	–	23.8	−37.9
	EPSR (Gaussian fit)^a^			21.9	−40.5

Values for *φ_H_* and *ψ_H_* were calculated from reported coupling constants using a Karplus-type relationship.[56] ^a^This work; ^b^lowest energy conformation for solvated cellobiose from French et al.[27]

On the whole, the EPSR averages for *φ_H_* and *ψ_H_* (Fig. 3) agree with the values obtained from current and previous investigations, with the cellobiose molecules adopting a *syn*-*φ_H_*/*ψ_H_* conformation in solution. The best agreement with previous measures is for the *ψ_H_* torsion,[16], [64] and, interestingly, the *φ_H_* value is in better agreement with recent DFT studies of solvated cellobiose,[27] compared with other previous measures. This is in opposition to recent vibrational spectroscopy measurements on the mircrohydration of phenyl β-cellobioside (which has a phenyl group in place of the hydrogen on the O1 oxygen on the non-reducing ring (Fig. 1)) in the gas phase where the molecules adopt a *syn*-*φ_H_*/*anti-ψ_H_* conformation.[28] The NMR-derived torsional constraints for *φ_H_* and *ψ_H_* maintained the torsions of the glycosidic linkage in the EPSR model of the neutron diffraction data in the *syn*-*φ_H_*/*ψ_H_* conformational state. Fig. 3 shows a fairly tight distribution for each measured angle with the exception of the *φ_H_* torsion of α-cellobiose that populated a second conformation (approximately 12% of the α-molecules; Fig. 3). This range of torsions shows that the EPSR model reproduced both the well-defined average glycosidic conformation and the flexibility characteristic of this linkage.

**Figure 4:**
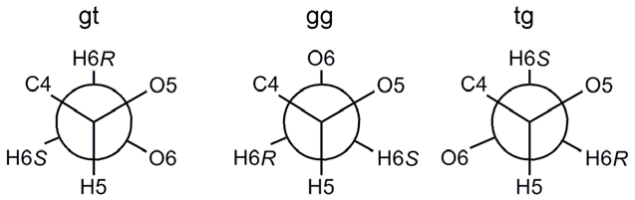
Newman projections of preferred hydroxymethyl conformations. The ω(′) torsion is viewed down the C5–C6 bond.

**Figure 5:**
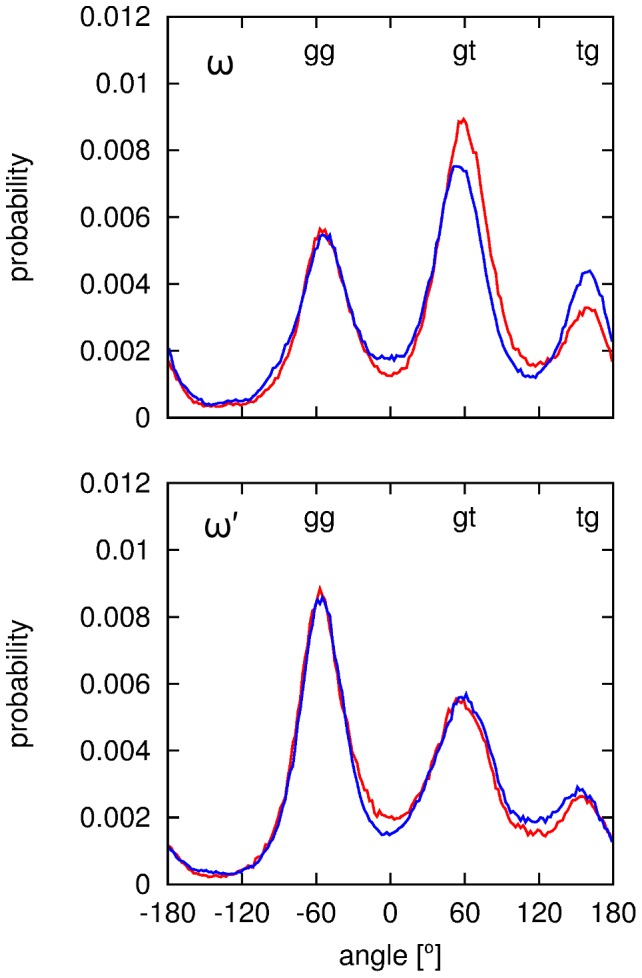
Distribution of hydroxymethyl conformations assumed by cellobiose in the EPSR model of the neutron diffraction data. Conformations of α-cellobiose are shown in red while those from β-cellobiose are shown in blue.

Cellobiose also has preferred hydroxymethyl orientations (ω(′); Fig. 2) in solution, as shown by the Newman projections for these conformations in Fig. 4. The three staggered orientations are generally referred to as gauche–trans (*gt*, ω = +65°), gauche–gauche (*gg*, ω = −65°) and trans–gauche (*tg*, ω = ±180°) due to the orientations of O5 and C4 relative to O6. Previous NMR studies of glucose,[65] glucose derivatives,[66] and cellobiose[54] have established the relative conformational distribution as P_gt_≈P_gg_>P_tg_ in aqueous solution, which is opposite to the P_tg_ conformation in the crystal structure of cellobiose[58] and the P_tg_ >> P_gg_≈P_gt_ distribution observed for native cellulose polymorphs.[6] Figure 5 shows the population distribution of hydroxymethyl conformations from the EPSR model, and Table 2 shows the calculated relative populations for each of the *gt*, *gg* and *tg* conformers compared with current NMR measurements and previous investigations of glucose[65] and cellobiose[54] in aqueous solutions.

**Table 2:** Comparison of hydroxymethyl rotamer populations derived from NMR studies and from the EPSR model.

	*ω* rotamer populations			*ω*' rotamer populations		
	P_gg_	P_gt_	P_tg_	P_gg_	P_gt_	P_tg_
α-d-glucose[65]	56	44	0	–	–	–
β-d-glucose[65]	53	45	2	–	–	–
α-cellobiose[54] ^,^ a	53	38	9	39	52	9
β-cellobiose[54] ^,^ a	48	44	8	38	53	9
α-cellobiose^b^	54	34	12	42	53	5
β-cellobiose^b^	52	42	6	42	53	5
α-cellobiose EPSR	31	51	18	47	39	14
β-cellobiose EPSR	33	45	22	42	39	19

a Values calculated from reported coupling constants using the method described by Olsson et al.[17]; ^b^This work (15 mM cellobiose).

The EPSR rotamer populations are in broad agreement with those from NMR studies, with the *tg* rotamer being the least populated and the *gg* and *gt* rotamers showing proportionally higher populations. This is consistent with prior NDIS/MD investigations of glucose in aqueous solution where a predominance of the *gt* and *gg* conformations were also observed.[44] Compared with previous and current NMR measurements–even at the slightly higher concentration measured here–EPSR gives the opposite trend for *gg* and *gt* rotamers where specifically the relative concentrations from EPSR are P_gt_>P_gg_>>P_tg_ compared with P_gg_>P_gt_>>P_tg_ from NMR measurements for ω and the order P_gg_>P_gt_>>P_tg_ for ω′ compared with P_gt_>P_gg_>>P_tg_ observed by NMR spectroscopy. For ω, the higher population of *gt* compared with *gg* is evident in Fig. 5; however, each rotamer gives a fairly broad distribution of angles around each orientation in the EPSR fits to the neutron data for both α- and β-cellobiose.

The variance between the NMR and the neutron diffraction data in ω′ appears to be due to the *tg* rotamer being slightly more populated in the EPSR model, as P_gg_ is similar from both the NMR and EPSR (Table 2). However, observation of the relative ω′ rotamer populations in Fig. 5 indicates this discrepancy may due to the fact that EPSR gives a distribution of rotamers, while NMR rotamer population assignment is via coupling constants and only gives a single averaged value. As a result, when assessing a distribution of rotamer angles from EPSR, the exact number of hydroxymethyl groups in any one given orientation is not always clear as the distributions can be broad, as is the case for the *tg* rotamer for the ω′ torsion in Fig. 5. Moreover the NMR results reported here are from the less concentrated sample as attempts to measure the higher concentration (0.88 M) led to spectra with poor resolution and as such the relative hydroxymethyl rotamer populations could not be accurately determined. It is possible that with a higher concentration of cellobiose in solution these rotamers have slightly different populations which are observed in the EPSR model. These results and those for NDIS studies on glucose[44] support the importance of carbohydrate–water hydrogen bonding in establishing the conformation of the hydroxymethyl groups as has been suggested by spectroscopic[66] and quantum- and molecular mechanics studies[67] since NDIS is particularly sensitive to hydrogen-bonding interactions.

### Cellobiose-Cellobiose Interactions

**Figure 6:**
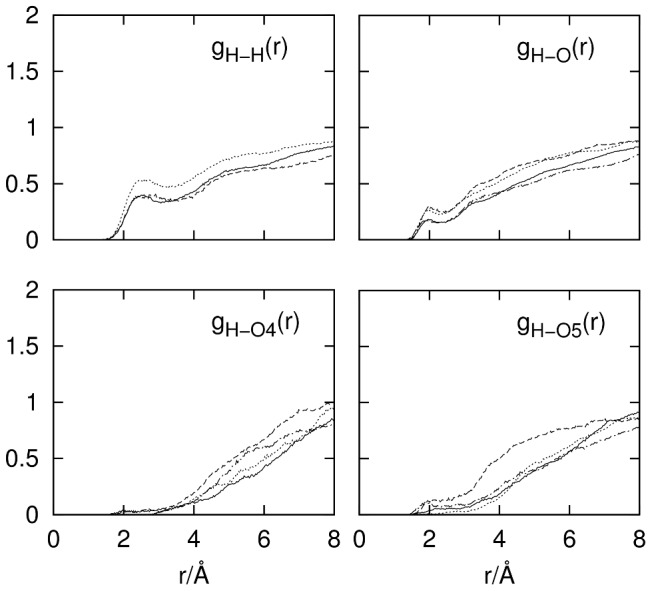
RDFs for intermolecular cellobiose–cellobiose interactions. For g_H–H_(r), the solid line represents interactions between hydroxyl protons both on reducing rings, the dashed line represents interactions between hydroxyl protons both on non-reducing rings, and the dotted line represents interactions between hydroxyl protons on reducing and non-reducing rings. For g_H–O_(r) the interaction between hydroxyl atoms both on reducing rings (solid), reducing ring hydrogen and non-reducing ring oxygen (dashed), non-reducing ring hydrogen and reducing ring oxygen (dotted) and atoms both on non-reducing rings (dash-dotted) are shown. The RDFs for g_H–O5_(r) follow the same line styles. For g_H–O4_(r), interactions between reducing ring hydrogens and O4 on α and β anomers are shown as solid and dashed lines, respectively, while the same are shown for non-reducing ring hydrogens as dotted and dash-dotted lines, respectively.

Figure 6 shows the RDFs for cellobiose–cellobiose interactions that might occur in solution. The RDFs in this figure are only shown for possible hydrogen-bonding interactions between different cellobiose molecules in solution. The other putative non-hydrogen bonding association would be via C–H interactions with atoms on other cellobiose molecules. The RDFs for these intermolecular interactions were virtually non-existent (see Supporting Information File S1). As is clear from Fig. 6, the intermolecular hydrogen-bonding interactions are very slight, indicating that there are very few interactions between individual cellobiose molecules even in these concentrated solutions. There are only very small peaks in both the H–H and O–H functions which show potential hydrogen bonding between –OH groups on the different molecules signifying very limited interactions. For instance, the coordination number of the small peak at ∼2Å in the O–H RDFs is 0.01 in each case. This indicates that only about 1% of the molecules show any hydrogen bonding between their OH groups. The H–H RDF shows slightly higher coordination with 8% of these molecules being associated in the solutions. Given the high concentration of molecules in solution, this is likely due to random interactions rather than any significant association of these molecules in this solution. The ring and linkage oxygens (C–O–C) to OH hydrogen RDFs (bottom panels Fig. 6) show even less association with no distinct peaks being present in these RDFs.

### Cellobiose Hydroxyl Group–Water Hydrogen Bonding

**Figure 7:**
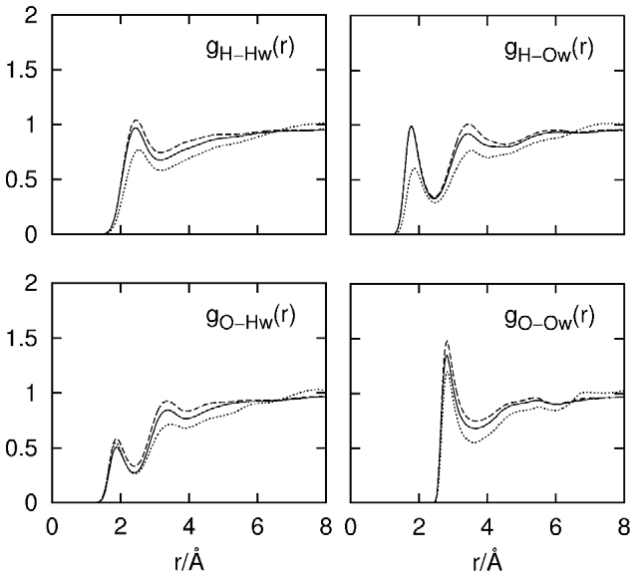
RDFs for cellobiose hydroxyl group–water interactions. Solid lines represent the non-reducing ring hydroxyl atoms. Dashed lines represent the reducing ring hydroxyl atoms. Dotted lines represent the atoms of the HO3 hydroxyl group.

Hydrogen-bonding interactions between cellobiose hydroxyl groups and water are easily distinguished via the RDFs from the hydroxyl group–water pairs (Fig. 7). The RDFs in this figure are averages from both cellobiose anomers with the contributions weighted according to the 40∶60 α-:β-cellobiose anomeric ratio. The only exceptions to this anomeric weighting are the RDFs for O4–X interactions where these atoms were labeled distinctly for α and β anomers in the EPSR model due to simulation routine requirements.

The cellobiose hydroxyl groups show similar hydration with respect to both oxygen and hydrogen atoms with the exception of the HO3 group where fewer hydrogen bonds are donated to water (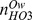
≈0.6). Integration of *g_H(')-Ow_*(*r*) from 0–2.46 Å shows an average of ∼0.8 hydrogen bonds being donated from cellobiose to water. Similar integration of *g_O(')-Hw_*(*r*) over the same distance range (0–2.46 Å) gives 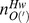
≈0.9–1.0 for all hydroxyl groups, showing that each of the hydroxyl oxygens accept, on average, one hydrogen bond from water. These results are consistent with earlier predictions for monosaccharides in aqueous solution where it was hypothesized that two hydrogen bonds per hydroxyl group are formed with the surrounding water solvent due to the strong downfield shifts of saccharide hydroxyl group NMR signals that indicate hydrogen bonding involving both the O and H atoms.[66] Furthermore, from an observed absence of both distinct saccharide υ_OH_ bands and water υ_OH_ shifts in infrared spectra, it has been suggested that water is both a donor and acceptor of hydrogen bonds from the hydroxyl group.[68]

### Cellobiose O4– and O5–Water Hydrogen Bonding

**Figure 8:**
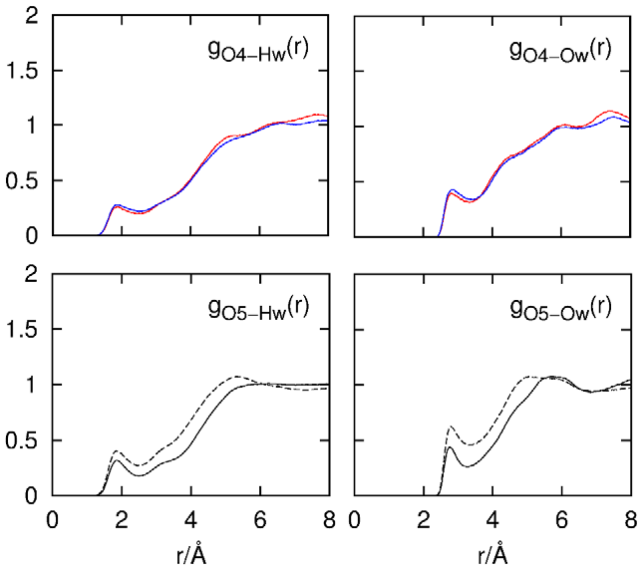
RDFs for interactions between the C–O–C oxygen atoms and water. The red and blue lines represent the glycosidic linkage oxygen (O4)–water interactions from α- and β-cellobiose, respectively. The solid and dashed lines represent the non-reducing oxygen (O5′)–water correlations and reducing ring oxygen (O5) atom–water correlations, respectively.

The glycosidic linkage oxygen atom–water RDFs for both α-cellobiose and β-cellobiose oxygens (O4α and O4β; Fig. 1) as well as the RDFs for water-oxgen interactions for both reducing and non-reducing ring oxygens are shown in Fig. 8. Both the glycosidic linakage oxygen and the non–reducing ring C–O–C oxygens accept fewer hydrogen bonds from water compared with the cellobiose hydroxyl groups. Integration of *g_O4-Hw_*(*r*) (0–2.46 Å) gives ∼0.6–0.7 hydrogen bonds, and similar integration of *g_O5'Hw_(r)* gives roughly the same number of bonds. This is similar to previous studies that have shown similar *under-*hydration of X–O–X (X ≠ H) when compared to hydroxyl, carbonyl or carboxylate groups in water.[43], [46], [48]

Intriguingly, the RDFs for the O5(′)–Hw and O5(′)–Ow atom pairs (Fig. 8, lower panel) show a decrease in the hydration of the non-reducing ring oxygen at 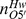
≈0.7 and 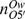
≈0.7 when compared to the reducing ring oxygens at 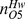
≈0.9 and 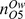
≈1.2. This reduced hydration of the non-reducing ring oxygen O5′ taken with similar reduced hydrogen-bond donation to water by HO3 suggests the population of an intramolecular O5′···HO3 hydrogen bond which has been predicted by DFT calculations, both in the presence and absence of solvation,[26], [27] by MD simulations[23] on cellobiose and by NMR spectroscopy of methyl α-cellobioside.[17] This is in opposition to gas phase microhydrated structures where there no O5′···HO3 hydrogen bonding interactions were observed but rather the cellobioside structure was stabilized by water-mediated O6′···HO3 interactions[28] and previous NMR/MD investigations on methyl β-cellobioside where it was concluded that an O5′···HO3 hydrogen bond was unlikely in water.[30]

**Figure 9:**
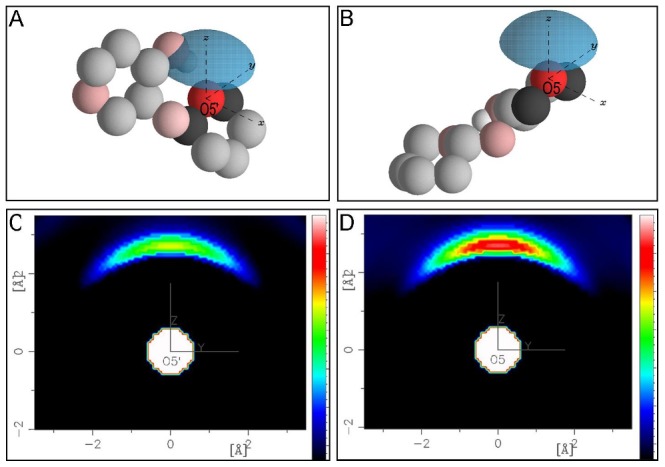
The distribution of water molecules about (A) O5′ and (B) O5 over a distance range of 0 –**3 Å.** Approximately 80% of water molecules around O5(′) are plotted in the blue distrubution shells surrounding the O5(′) oxygens on the origin of the central axis. Additional atoms from the cellobiose molecule are plotted (in lighter shades) but are not representative of the actual average conformation of cellobiose molecules in the EPSR model. The atoms are shown for clarity and context. Two-dimensional projections of the SDFs onto the *yz* plane are shown in C and D where the relative intensity scales (color bars) are identical.

Given the differences observed in the reducing and non-reducing ring O5 and O5′–water RDFs, the spatial density functions (SDFs) for the distributions of water around O5 and O5′ were calculated from the EPSR modeling box. SDFs (Figs. 9A–B) give the most probable *location* in 3-dimensions of water molecules around the C1′–O5′–C5 fragments of cellobiose in solution. Specifically in Figs. 9A-B the cellobiose O5′ atom is placed at the center of the laboratory axis, and the distribution of Ow atoms around these atoms is shown as a blue probability shell. Additional atoms from cellobiose are plotted to aid in visually orienting the relevant C–O–C fragment, but the atomic coordinates do not represent the average conformation of cellobiose in the EPSR simulation. The SDFs are averaged over the α:β anomeric distribution, and mathematical details of the spherical harmonic expansion calculation are given in the Supporting Information File S1. In Figs. 9A–B, the blue shells represent the most probable location of 80% of the water molecules within 0–3.0 Å of O5′ and O5, respectively. For both O5 and O5′ the water molecules are located predominantly in the positive *z*-direction above the cellobiose molecules with an absence of density for water in the *xy*-plane.

The 3D SDF of water around the O5′ and O5 atoms do not give the total number of water molecules present in the surrounding blue shells in Fig. 9, but rather give the highest probability of finding a water molecule at these distances. Although the absolute number of waters in these shells cannot be precisely determined, a cross-sectional projection of these SDFs can demonstrate the relative number of water molecules in the probability shells of one oxygen compared with the other. Figures 9C–D display these 2D projections onto the *yz*-plane and show that the distribution of waters around the O5′ (Fig. 9C) is reduced in intensity relative to waters around the reducing-ring oxygen (O5; Fig. 9D), indicating that there are slightly more water molecules present around the O5 oxygen compared to the non-reducing ring oxygen. Steric hindrance of O5′ hydration, given its proximity to the glycosidic linkage, could, in part, be responsible for the reduced spatial extent of the hydration shell compared with that for O5 (Fig. 9D). However, the reduced presence of water along the defined *z*-axis again suggests competition between water and HO3 in forming hydrogen bonds with O5′.

### Cellobiose Intramolecular Hydrogen Bonding

**Figure 10:**
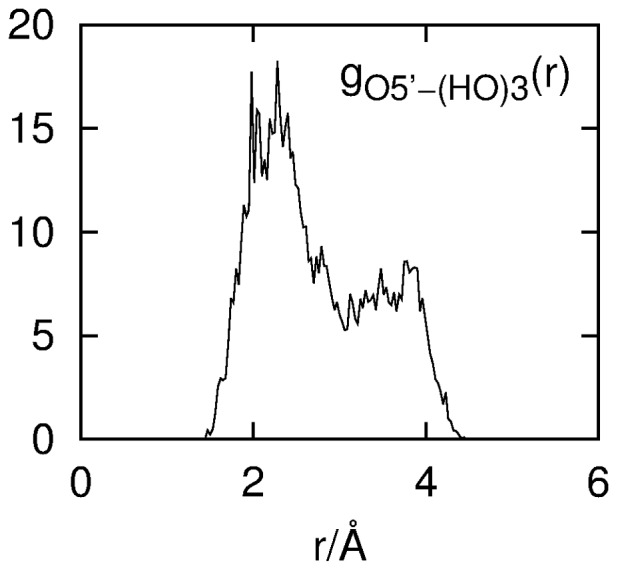
Intramolecular RDF corresponding to the O5′···HO3 hydrogen bond.

**Figure 11:**
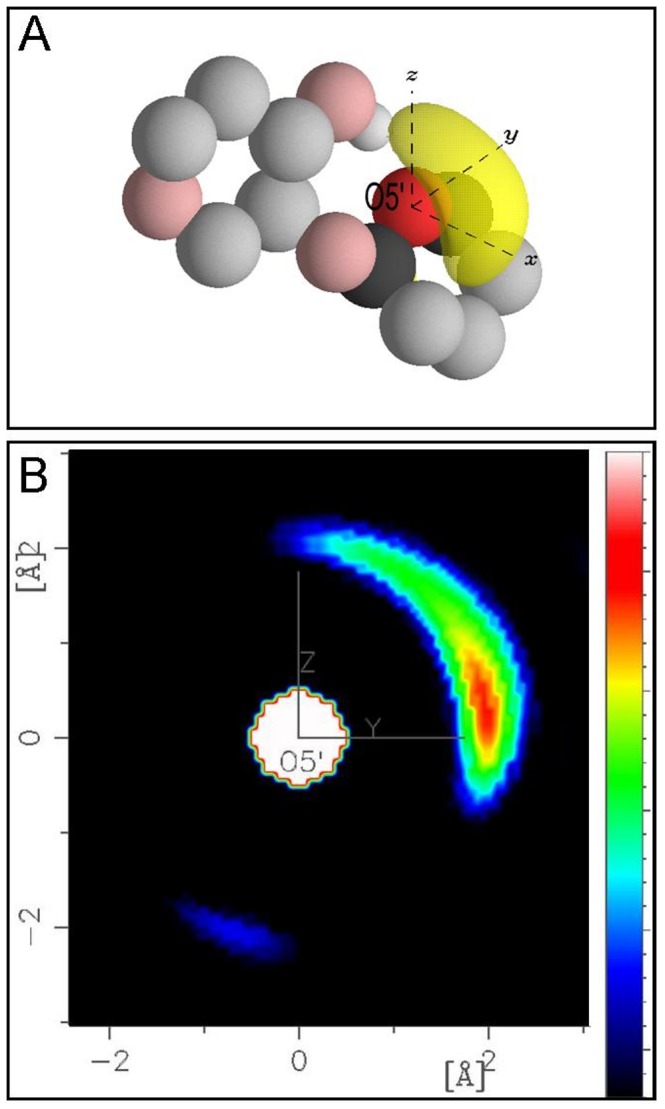
Distribution of HO3 about O5′. (A) Approximately 25% of the hydroxyl hydrogen (H3) locations over a distance range of 1.5–3.0 Å around O5′ at the origin are shown are shown. Additional atoms from cellobiose are plotted for context as in Fig. 9A–B (B) A two-dimensional projection of the SDF from (A) onto the *yz* plane is shown. The intensity scale (color bar) is not the same as that used in Fig. 9C–D.

Figure 10 shows the RDF for the O5′–HO3 atom pair for both α- and β-cellobiose. The broad peak with a maximum at 2.28 Å is consistent with an O5′···HO3 hydrogen bond; however, the distance range of this peak (∼1.5–3.1 Å) shows that this hydrogen-bonding interaction is less well defined than the O5′···Hw water hydrogen bond whose corresponding RDF peak is considerably sharper (Fig. 7). Previous DFT studies of solvated cellobiose predict a similar distance for this intramolecular bond ranging from 1.93 to 2.48 Å.[27] Integration of *g_O5'-HO3_*(*r*) curve (Eq. 3) in Fig. 10 gives an intramolecular coordination number of ∼0.46 indicating that this hydrogen bond is populated approximately 50% of the time when cellobiose is in aqueous solution. Similar to the water SDF around O5′ in Fig. 9A, Fig. 11A shows the SDF for the most probable *location* of HO3 atoms around O5′ with the O5′ on the central axis, where in this figure the yellow shell represents the most probable 25% of HO3 locations around O5′–HO3 at a distance of 1.5–3.0 Å—corresponding to the minimum and maximum distance in the RDF peak from Fig. 10. Again, similar to Figs 9 A–B, the cellobiose molecule is plotted only for clarity and to guide the reader, and as such does not represent the average orientation of cellobiose molecules in the solution. In Fig. 11, the HO3 hydroxyl group is generally located in the positive *xy* plane in a distribution consistent with rotations about the C3–O3 bond and conformational flexibility about the glycosidic linkage. Analogous to Fig. 9C the 2D projection of this SDF onto the *yz* plane (Fig. 11B) clearly shows that this hydroxyl group overlaps with the O5′ hydration shell (Fig. 9C) thus contributing to the reduced hydration of O5′.

## Conclusions

The structure of cellobiose in aqueous solution has been investigated with a combination of structural techniques–NMR spectroscopy and NDIS augmented with EPSR computer modeling. Using this combination of experimental and computational techniques an atomic-level structure of cellobiose *in solution* has been determined that most notably gives firm evidence of the intramolecular hydrogen bond between the non-reducing ring O5′ and the reducing ring HO3 group. The existence of this O5′···HO3 hydrogen bond has been previously predicted by NMR spectroscopic studies on methyl α-cellobioside[17] in aqueous solution, and this bond has also been shown to be persistent for the same molecule in DMSO solvent.[18] The existence of this bond has also been determined by computational studies[25]–[27] and importantly in DFT investigations which are compared directly with NMR coupling constant measurements. Conversely, other NMR investigations on methyl β-cellobioside in both water and in methanol/water solutions concluded that the existence of any O5′···HO3 bonds were “insignificant”.[29], [30] Although both methyl β-cellobioside and methyl α-cellobioside are slightly different than cellobiose measured here, in that both have an -OCH_3_ group (fixed respectively in the β- or α- position on C1) instead of an -OH on the C1 carbon atom, which is free to mutarotate (Fig. 1), it is unlikely this methoxy substitution would have much effect on the existence of the intramolecular hydrogen bond between the O5′ and HO3 group in solution.

Diffraction techniques provide a *direct* determination of the structure of present in solution as opposed to spectroscopies which can only infer structure, as these techniques only directly measure the dynamical aspects of molecules in the system. Here the measured NDIS data, interpreted through use of EPSR simulation, provide a physically reasonable model of the structure of cellobiose in water that is consistent with both neutron data and the NMR experiments. Previous findings on similar carbohydrate molecules noted that molecular geometry was ‘dictated’ by inter-residue hydrogen bonding.[19] This is consistent with the present work; the O5′···HO3 bond in the EPSR model was reproduced only by virtue of constraints to the average glucose ring conformations and a single non-bonding distance across the glycosidic linkage. NDIS techniques are particularly useful as they can also quantify the average coordination observed in a liquid. Importantly, the neutron data is not only consistent with the presence of an inter-residue O5′•••HO3 bond, the hydrogen bonding to the reducing ring O5′ oxygen is shared between water and the HO3 hydroxyl group with an average of 50% occupancy by each hydrogen-bond donor.

Other potential water hydrogen-bonding sites, namely the hydroxyl groups and ether oxygen atoms in the cellobiose molecule, have also been assessed. It was found that, on the average, each hydroxyl hydrogen donates ∼0.8 hydrogen bonds to water with the exception of the HO3 group, which shows a relatively smaller number of H–Ow interactions of 0.6 hydrogen bonds to water. The C–O–C oxygen atoms–both the glycosidic linkage and the ring oxygens–accept on the average ∼0.7 Hw–O hydrogen bonds from water, with the reducing ring oxygen (O5; Fig. 1) showing a slightly larger hydration of ∼0.9 accepted from the surrounding water solvent. This reduction in hydration from the glycosidic linkage oxygen and the non-reducing ring oxygen are reflective of the O5′ and HO3 hydrogen bonding interaction as the presence of this bond reduces the number of water molecules that may bind to either of these oxygen atoms in cellobiose.

The conformational aspects of the cellobiose structure are also delineated, both from NMR and from the EPSR model of the neutron diffraction data. The glycosidic torsion angles *φ_H_* and *ψ_H_* from the neutron data show a fairly tight distribution of angles around approximately 22^°^ and −40^°^, respectively, *syn*-*φ_H_*/*ψ_H_* or ‘trans’ in solution, consistent with NMR measurements here and those previously reported,[16], [64] as well as DFT studies.[27] Similarly hydroxymethyl torsional angles for both reducing and non-reducing rings from the neutron diffraction measurements are broadly consistent with the NMR data measured here as well as for previous measures of cellobiose in solution.[54]

## Supporting Information

File S1 **Supporting Information.**
(DOC)

## References

[1] Kamm B, Kamm M, Gruber PR, Kromus S (2006) Biorefinery Systems–An Overview. In: Kamm B, Gruber PR, Kamm M, editors. Biorefineries–Industrial Processes and Products. Weinheim: Wiley-VCH. 3–33.

[2] Sponsler OL, Dore WH (1926) The structure of ramie cellulose as derived from X-ray data. Fourth Colloid Symposium Monograph 41: 174–202.

[3] Meyer KH, Mark H (1928) Uber den Bau des krystallisierten Anteils der Cellulose. Ber Dtsch Chem Ges 61B: 593–614.

[4] Meyer KH, Misch H (1936) Positions des atomes dans la nouveau modele spacial de la cellulose. Helv Chim Acta 20: 232–244.

[5] Atalla RH, VanderHart DL (1984) Native Cellulose: A Composite of Two Distinct Crystalline Forms. Science 223: 283–285. 17801599 10.1126/science.223.4633.283

[6] O'Sullivan AC (1997) Cellulose: the structure slowly unravels. Cellulose 4: 173–203.

[7] Zugenmaier P (2008) Crystalline Cellulose and Derivatives; Timell TE, Wimmer R, editors. Heidelberg: Springer.

[8] Flugge LA, Blank JT, Petillo PA (1999) Isolation, Modification, and NMR Assignments of a Series of Cellulose Oligomers. J Am Chem Soc 121: 7228–7238.

[9] Missori M, Mondelli C, De Spirito M, Castellano C, Biccheri M, et al (2006) Modifications of Mesoscopic Structure of Cellulose in Paper Degradation. Phys Rev Lett 97: 2380011–2380014. 10.1103/PhysRevLett.97.238001 17280248

[10] De Spirito M, Mauro M, Massimiliano P, Giueseppe M, Jose T, et al (2008) Modifications in solvent clusters embedded along the fibers of a cellulose polymer network cause paper degradation. Phys Rev E: Stat Phys, Plasmas, Fluids 77: 41801. 10.1103/PhysRevE.77.041801 18517646

[11] Sugiyama M, Iigima H, Hara K, Nakamura A, Hiramatsu N, et al (2000) Structural investigation on aqueous suspension of microcrystalline cellulose. TMRSJ 25: 743–746.

[12] Nishiyama Y, Langan P, Chanzy H (2002) Crystal Structure and Hydrogen-Bonding System in Cellulose Iβ from Synchrotron X-ray and Neutron Fiber Diffraction. J Am Chem Soc 124: 9074–9082. 12149011 10.1021/ja0257319

[13] Nishiyama Y, Sugiyama J, Chanzy H, Langan P (2003) Crystal structure and hydrogen bonding system in cellulose Iα from synchrotron X-ray and neutron fiber diffraction. J Am Chem Soc 125: 14300–14306. 14624578 10.1021/ja037055w

[14] Parfondry A, Cyr N, Perlin AS (1977) C-13-H-1 Inter-Residue Coupling in Disaccharides, and the Orientations of Glycosidic Bonds. Carbohydr Res 59: 299–309.

[15] Sugiyama H, Hisamichi K, Usui T, Sakai K, Ishiyama J (2000) A study of the conformation of β-1,4-linked glucose oligomers, cellobiose to cellohexaose, in solution. J Mol Struct 556: 173–177. 10.1016/s0008-6215(99)00310-9 10795808

[16] Cheetham NWH, Dasgupta P, Ball GE (2003) NMR and modelling studies of disaccharide conformation. Carbohydr Res 338: 955–962. 12681919 10.1016/s0008-6215(03)00069-7

[17] Olsson U, Serianni AS, Stenutz R (2008) Conformational Analysis of β-Glycosidic Linkages in ^13^C Labeled Glucobiosides Using Inter-residue Scalar Coupling Constants. J Phys Chem B 112: 4447–4453. 18345660 10.1021/jp710977k

[18] Zhang W, Zhou H, Carmichael I, Serianni AS (2009) An NMR investigation of putative interresidue H-bonding in methyl α-cellobioside in solution. Carbohydr. Res., 344: , 1582–1587. 10.1016/j.carres.2009.06.007 19632671

[19] Zhou H, Pan Q, Zhang W, Carmichael I, Serianni AS (2007) DFT and NMR studies of ^3^ *J* _COH_, ^3^ *J* _HCOH_, and ^3^ *J* _CCOH_ Spin-Couplings in Saccharides: C–O Torsional Bias and H-bonding in Aqueous Solutions. J. Org. Chem. 72: , 7071–7082. 10.1021/jo0619884 17316047

[20] Poppe L, van Halbeek H (1994) NMR spectroscopy of hydroxyl protons in supercooled carbohydrates. Nat Struct Biol 1: 215–216. 7656046 10.1038/nsb0494-215

[21] Bernet B, Vasella A (2000) Intra- and Intermolecular H-Bonds of Alcohols in DMSO H-1 NMR Analysis of Inter-Residue H-Bonds in Selected Oligosaccharides: Cellobiose, Lactose, N,N'Diacetylchitobiose, Maltose, Sucrose, Agarose, and Hyaluronates. Helv Chim Acta 83: 2055–2072.

[22] Rees DA, Skerrett RJ (1968) Conformational Analysis of Cellobiose, Cellulose, and Xylan. Carbohydr Res 7: 334–348.

[23] Pereira CS, Kony D, Baron R, Muller M, van Gunsteren WF, et al (2006) Conformational and dynamical properties of disaccharides in water: a molecular dynamics study. Biophys J 90: 4337–4344. 16581848 10.1529/biophysj.106.081539 PMC1471844

[24] Umemura M, Yuguchi Y, Hirotsu T (2005) Hydration at glycosidic linkages of malto- and cello-oligosaccharides in aqueous solution from molecular dynamics simulation: Effect of conformational flexibility. J Mol Struct 730: 1–8.

[25] Christensen NJ, Hansen PI, Larsen FH, Folkerman T, Motawia MS, et al (2010) A combined nuclear magnetic resonance and molecular dynamics study of the two structural motifs for mixed-linkage beta-glucans: methyl beta-cellobioside and methyl beta-laminarabioside. Carbohydr Res 345: 474–486. 20079487 10.1016/j.carres.2009.12.009

[26] French AD, Johnson GP (2006) Quantum mechanics studies of cellobiose conformations. Can J Chem 84: 603–612.

[27] French AD, Johnson GP, Cramer CJ, Csonka GI (2012) Conformational analysis of cellobiose by electronic structure theories. Carbohydr Res 350: 68–76. 22265378 10.1016/j.carres.2011.12.023

[28] Pincu M, Cocinero EJ, Mayorkas N, Brauer B, Davis BG, et al (2011) Isotopic Hydration of Cellobiose: Vibrational Spectroscopy and Dynamical Simulations. J Phys Chem A 115: 9498–9509. 21631124 10.1021/jp112109p

[29] Leeflang BR, Vliegenthart JFG, Kroon-Batenburg LMJ, van Eijck BP, Kroon J (1992) A Proton NMR and MD study of intramolecular hydrogen bonds in methyl β–cellobioside. Carbohydr Res 230: 41–61. 1511454 10.1016/s0008-6215(00)90512-3

[30] Kroon J, Kroon-Batenburg LMJ, Leeflang BR, Vliegenthart JFG (1994) Intramolecular versus intermolecular hydrogen bonding in solution. J Mol Struct 322: 27–31.

[31] McLain SE, Benmore CJ, Siewenie JE, Urquidi J, Turner JFC (2004) On the structure of liquid hydrogen fluoride. Angew Chem Int Ed 43: 1952–1955. 10.1002/anie.200353289 15065271

[32] Soper AK, Ricci MA (2000) Structures of high-density and low-density water. Phys Rev Lett 84: 2881–2884. 11018966 10.1103/PhysRevLett.84.2881

[33] Bowron D, Finney J, Soper AK (2006) The structure of liquid tetrahydrofuran. J Am Chem Soc 128: 5119–5126. 16608347 10.1021/ja0583057

[34] Venturi G, Formisano F, Cuello GJ, Johnson MR, Pellegrini E, et al (2009) Structure of liquid n-hexane. J Chem Phys 131: 034508/1–034508/9. 19624210 10.1063/1.3176413

[35] Headen TF, Howard CA, Skipper NT, Wilkinson MA, Bowron DT, et al (2010) Structure of π–π Interactions in Aromatic Liquids. J Am Chem Soc 132: 5735–5742. 20102204 10.1021/ja909084e

[36] Imberti S, Bowron DT (2010) Formic and acetic acid aggregation in the liquid state. J Phys: Condens Matter 22: 404212/1–404212/14. 21386573 10.1088/0953-8984/22/40/404212

[37] Dixit S, Crain J, Poon WCK, Finney JL, Soper AK (2002) Molecular segregation observed in a concentrated alcohol–water solution. Nature 416: 829–832. 11976678 10.1038/416829a

[38] McLain SE, Soper AK, Luzar A (2007) Investigations on the structure of dimethyl sulfoxide and acetone in aqueous solution. J Chem Phys 127: 174515/1–174515/12. 17994835 10.1063/1.2784555

[39] Deetlefs M, Hardacre C, Nieuwenhuyzen M, Sheppard O, Soper AK (2005) Structure of ionic liquid–benzene mixtures. J Phys Chem B 109: 1593–1598. 16851130 10.1021/jp047742p

[40] Mancinelli R, Botti A, Bruni F, Ricci MA, Soper AK (2007) Perturbation of water structure due to monovalent ions in solution. PCCP 9: 2959–2967. 17551619 10.1039/b701855j

[41] Mancinelli R, Botti A, Bruni F, Ricci MA, Soper AK (2007) Hydration of sodium, potassium, and chloride ions in solution and the concept of structure maker/breaker. J Phys Chem B 111: 13570–13577. 17988114 10.1021/jp075913v

[42] Mason PE, Neilson GW, Enderby JE, Saboungi ML, Brady JW (2005) Structure of aqueous glucose solutions as determined by neutron diffraction with isotopic substitution experiments and molecular dynamics calculations. J Phys Chem B 109: 13104–13111. 16852630 10.1021/jp040622x

[43] Hulme EC, Soper AK, McLain SE, Finney JL (2006) The hydration of the neurotransmitter acetylcholine in aqueous solution. Biophys J 91: 2371–2380. 16798812 10.1529/biophysj.106.089185 PMC1557574

[44] Mason PE, Neilson GW, Enderby JE, Saboungi MLC, Cuello G, et al (2006) Neutron diffraction and simulation studies on the exocyclic hydroxymethyl conformation of glucose. J Chem Phys 125: 224505/1–224505/9. 17176147 10.1063/1.2393237

[45] McLain SE, Soper AK, Diadone I, Smith JC, Watts A (2008) Charged-Based Interactions between Peptides Observed as the Dominant Force for Association in Aqueous Solution. Angew Chem Int Ed 47: 9059–9062. 10.1002/anie.200802679 18937237

[46] Foglia F, Lawrence MJ, Lorenz CD, McLain SE (2010) On the hydration of the phosphocholine headgroup in aqueous solution. J Chem Phys 133: 145103(145101–145110).. 20950050 10.1063/1.3488998

[47] Malardier-Jugroot C, Bowron DT, Soper AK, Johnson ME, Head-Gordon T (2010) Structure and water dynamics of aqueous peptide solutions in the presence of co-solvents. Phys Chem Chem Phys12: 382–392. 10.1039/b915346b 20023816

[48] Pagnotta SE, McLain SE, Soper AK, Bruni F, Ricci MA (2010) Water and Trehalose: How Much Do They Interact with Each Other? J Phys Chem B 114: 4904–4908. 20297794 10.1021/jp911940h

[49] Hargreaves R, Bowron DT, Edler K (2011) Atomistic Structure of a Micelle in Solution Determined by Wide Q-Range Neutron Diffraction. J Am Chem Soc 133: 16524–16536. 21823595 10.1021/ja205804k

[50] Sears V (1992) Neutron scattering lengths and cross sections. Neutron News 3: 29–37.

[51] Soper AK (2005) Partial structure factors from disordered materials diffraction data: an approach using empirical potential structure refinement. Phys Rev B: Condens Matter 72: 104204–104216.

[52] Towey JJ, Soper AK, Dougan L (2011) Preference for Isolated Water Molecules in a Concentrated Glycerol–Water Mixture. J Phys Chem B 115: 7799–7807. 21612256 10.1021/jp203140b

[53] Soper AK, Howells WS, Hannon AC (1989) ATLAS–analysis of time-of-flight diffraction data from liquid and amorphous samples. ISIS Pulsed Neutron Source, Rutherford Appleton Laboratory.

[54] Roslund MU, Tahtinen P, Miemitz M, Sjoholm R (2008) Complete Assignments of the H-1 and C-13 chemical shifts and J H-H coupling constants in NMR spectra of D-glucopyranose and all D-glucopyranosyl-D-glucopyranosides. Carbohydr Res 343: 101–112. 17980865 10.1016/j.carres.2007.10.008

[55] Willker W, Leibritz D (1995) Determination of Heteronuclear Long-Range H,X Coupling Constants from Gradient-Selected HMBC Spectra. Magn Reson Chem 33: 632–638.

[56] Cloran F, Carmichael I, Serianni AS (1999) Density Functional Calculations on Disaccharide Mimics: Studies of Molecular Geometries and Trans-*O*-glycosidic *^3^J_COCH_* and *^3^J_COCC_* Spin-Couplings. J Am Chem Soc 121: 9843–9851.

[57] Stenutz R, Carmichael I, Widmalm G, Serianni AS (2002) Hydroxymethyl Group Conformation in Saccharides: Structural Dependencies of ^2^ *J* _H,H_, ^3^ *J* _H,H_ and ^1^ *J* _C,H_ Spin–Spin Coupling Constants. J Org Chem 67: 949–958. 11856043 10.1021/jo010985i

[58] Chu SSC, Jeffrey GA (1968) Refinement of crystal structures of β-D-glucose and cellobiose. Acta Crystallogr, Sect B: Struct Sci 24: 830–838.

[59] Shallenberger RS (1982) Advanced Sugar Chemistry: Principles of Sugar Stereochemistry. Westport, CT: AVI Publishing Co., Inc.

[60] Guvench O, Greene SN, Kamath G, Brady JW, Venable RM, et al (2008) Additive Empirical Force Field for Hexopyranose Monosaccharides. J Comput Chem 29: 2543–2564. 18470966 10.1002/jcc.21004 PMC2882059

[61] Shen T, Langan P, French AD, Johnson GP, Gnanakaran S (2009) Conformational Flexibilitiy of Soluble Cellulose Oligomers: Chain Length and Temperature Dependence. J Am Chem Soc 131: 14786–14794. 19824731 10.1021/ja9034158

[62] Berendsen HJC, Grigera JR, Straatsma TP (1987) The missing term in effective pair potentials. J Phys Chem 91: 6269–6271.

[63] IUPAC-IUB (1983) Symbols for Specifying the Conformation of Polysaccharide Chains. Pure Appl Chem 55: 1269–1272. 10.1111/j.1432-1033.1983.tb07224.x 6832145

[64] Sugiyama H, Hisamichi K, Usui T, Sakai K, Ishiyama JI (2000) A study of the conformation of β-1,4-linked glucose oligomers, cellobiose to cellohexaose, in solution. THEOCHEM 556: 173–177. 10.1016/s0008-6215(99)00310-9 10795808

[65] Nishida Y, Ohrui H, Meguro H (1984) H-1 NMR Studies of (6R)- and (6S)-Deuterated D-Hexoses: Assignemnet of the Preferred Rotamers about C5–C6 Bond of D-Glucose and D-Galactose Derivatives in Solutions. Tetrahedron Lett 25: 1575–1578.

[66] Rockwell GD, Grindley TB (1998) Effect of Solvation on the Hydroxymethyl Groups in Carbohydrates. J Am Chem Soc 120: 10953–10963.

[67] Barnett CB, Naidoo KJ (2008) Stereoelectronic and Solvation Effects Determine Hydroxymethyl Conformational Preferences in Monosaccharides. J Phys Chem B 112: 15450–15459. 18989909 10.1021/jp8067409

[68] Harvey J, Symnons M (1978) The Hydration of Monosaccharides–an NMR study. J Solution Chem 7: 571–586.

