# Supplementary material for: Structural Evidence for Inter-Residue Hydrogen Bonding Observed for Cellobiose in Aqueous Solution
Source: PLoS One. 2012 Oct 2;7(10):e45311. doi: 10.1371/journal.pone.0045311 (PMC3462749; doi:10.1371/journal.pone.0045311)
Supplement: File S1 — Supporting Information. (DOC) [file pone.0045311.s001.doc]

## Structural Evidence for Inter-Residue Hydrogen Bonding Observed for Cellobiose in Aqueous Solution—Supporting Information File S1

## William B. O’Dell, David C. Baker and Sylvia E. McLain

## Table of Contents

Spatial density functions from EPSR S2

Neutron diffraciton measurement details S3

NMR measurement details S4

EPSR model parameters S8

EPSR torsion angle constraints S9

Additional RDFs from EPSR fits to the neutron diffraction data S13

References S20

## The derivation of SDFs from EPSR

Having found, through EPSR, a model liquid structure consistent with the diffraction data, it is useful to extract structural information from the simulation box concerning the intermolecular distributions. Because the site–site RDFs only give a one-dimensional representation of the fluid, it is difficult to use these distances to visualize the local spatial and orientational order in three dimensions. For this reason, spatial density functions (SDF),[1,2] which allow a three-dimensional representation of the liquid structure to be constructed, were used to help determine the most probable nearest neighbor positions for both fluids in the present study. These tasks are achieved *via* spherical harmonic expansion of the full orientational pair correlation function,[3,4] using the simulation box to derive the positional and orientational coordinates of the molecules.

The details of the spherical harmonic expansion as well as the orientational correlation function calculation using a spherical harmonic expansion are given in detail elsewhere.[3,4] Here a summary of these techniques which follow the notation used by Gray and Gubbins explicitly is presented.[4]

A set of Euler angles within the laboratory reference frame $\omega_{M}\equiv(\phi_{M}\theta_{M}\chi_{M})$ for each molecule *M* is calculated using a predefined set of molecular coordinate axes. The corresponding set of generalized spherical harmonic functions, $D_{mn}^{l}\left(\omega_{M}\right)$, are calculated for each molecule and for a range of (*l,m,n*) values (up to *l* = 4 in the present instance). The set of such functions is then correlated taking into account the relative position $\mathbf{r}\equiv\left(r,\omega_{L}\right)\equiv\left(r,\theta_{L}\phi_{L}\right)$ of the second molecule with respect to the first, yielding a set of orientational correlation function expansion coefficients, $g(l_{1}l_{2}l;n_{1}n_{2};r)$.[4] From these coefficients the full orientational pair correlation function is obtained as an expansion of the form:

$g\left(\mathbf{r},\omega_{1},\omega_{2}\right)=\sum_{l_{1}l_{2}l} \sum_{m_{1}m_{2}m} \sum_{n_{1}n_{2}} g\left(l_{1}l_{2}l;n_{1}n_{2};r\right)C\left(l_{1}l_{2}l;m_{1}m_{2}m\right)D_{m_{1}n_{1}}^{l_{1}}\left(\omega_{1}\right)^{*}D_{m_{2}n_{2}}^{l_{2}}\left(\omega_{2}\right)^{*}D_{m0}^{l}\left(\omega_{L}\right)$ (1)

where $C\left(l_{1}l_{2}l;m_{1}m_{2}m\right)$ are the Clebsch–Gordan coefficients, $\omega_{1}$represents the Euler angles of molecule 1, $\omega_{2}$represents the Euler angles of molecule 2, and $\mathbf{r}=\left(r,\omega_{L}\right)$ represents the position of molecule 2 relative to molecule 1 in the laboratory coordinate frame.

In order to reconstruct the orientational correlation function it is convenient to set molecule 1 at the origin and orient it so that $\omega_{1}$= 0. This serves to define the coordinate system about which the spatial density and orientation of second (neighboring) molecules will be plotted. It also leads to an immediate simplification of equation (1) in that $D_{mn}^{l}\left(000\right)=\delta\left(mn\right)$, so that combining this with the requirement from the Clebsch–Gordan coefficients that $m=m_{1}+m_{2}$, the orientational pair correlation function relative to a central molecule at the origin is given by

$g\left(r,\omega,\omega_{M}\right)=\sum_{l_{1}l_{2}l} \sum_{m} \sum_{n_{1}n_{2}} g\left(l_{1}l_{2}l;n_{1}n_{2};r\right)C\left(l_{1}l_{2}l;n_{1}m_{2}m\right)D_{m_{2}n_{2}}^{l_{2}}\left(\omega_{M}\right)^{*}D_{m0}^{l}\left(\omega_{L}\right)$ (2)

where $m_{2}=m-n_{1}$. The spatial density function is generated by averaging the full orientational pair correlation function over the orientations of the second molecule, $\omega_{M}\equiv\left(\phi_{M}\theta_{M}\chi_{M}\right)$, which immediately eliminates any terms in the summation shown in equation (2) for which $l_{2},m_{2},n_{2}\neq0$. Hence the spatial density function is expressed as

$g\left(r,\omega\right)=\sum_{l_{1}} \sum_{n_{1}} g\left(l_{1}0l_{1};n_{1}0;r\right)C\left(l_{1}0l_{1};n_{1}0n_{1}\right)D_{n_{1}0}^{l_{1}}\left(\omega_{L}\right)$ (3)

from the closure relations for the Clebsch–Gordan coefficients ($l_{1}+l_{2} \geq l \geq\left|l_{1}-l_{2}\right|$).

## Neutron diffraction measurement details

Table S1 lists the samples measured by neutron diffraction on SANDALS where –(OH) and –(OD) describe the exchange at the solute hydroxyl groups. The sample preparation is described in the main text. Figure S1 shows the resultant diffraction patterns (*F(Q)*) along with the EPSR fits to the data (described below and in the main text).

**Table S1**. Isotopomers of cellobiose–water solutions measured by NDIS.

|  | solute | %D_2_O | %H_2_O |
| --- | --- | --- | --- |
| I | cellobiose-(OH) | — | 100 |
| II | cellobiose-(OD)_0.25_(OH)_0.75_ | 25 | 75 |
| III | cellobiose-(OD)_0.5_ (OH)_0.5_ | 50 | 50 |
| IV | cellobiose-(OD)_0.625_(OH)_0.375_ | 62.5 | 37.5 |
| V | cellobiose-(OD)_0.75_(OH)_0.25_ | 75 | 25 |
| VI | cellobiose-(OD)_0.875_(OH)_0.125_ | 87.5 | 12.5 |
| VII | cellobiose-(OD) | 100 | — |

Roman numerals correspond to structure factors plotted in Figure S1.


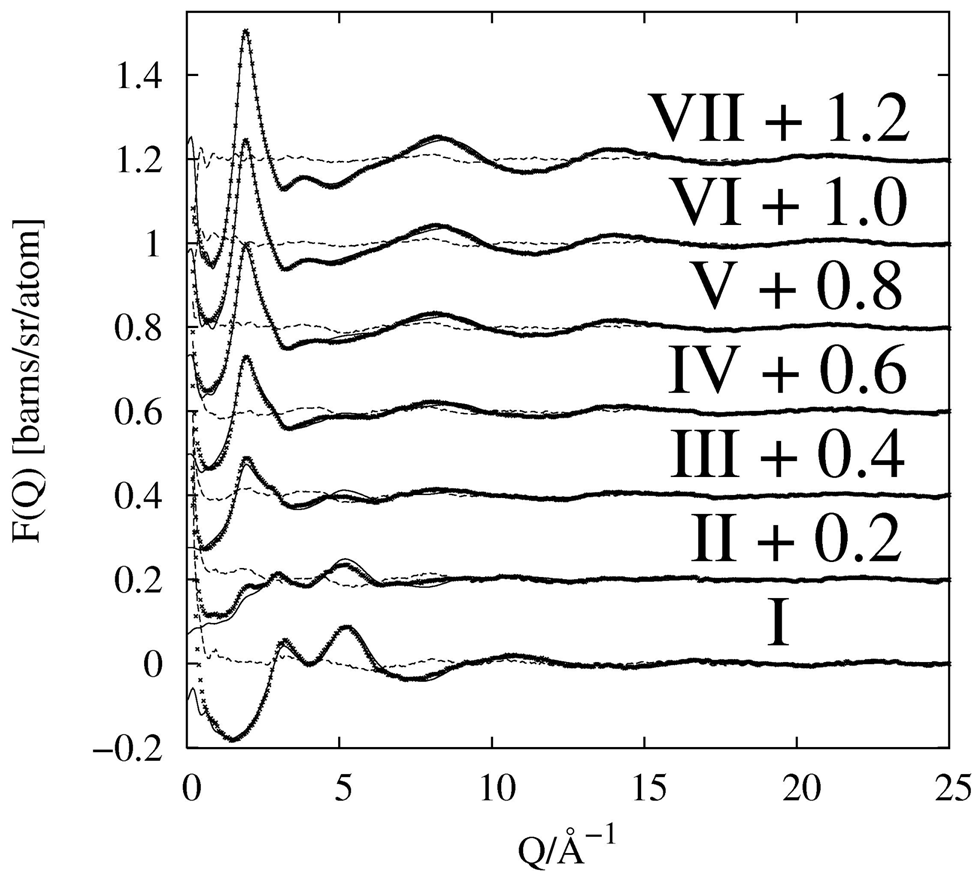


**Figure S1**. Neutron diffraction data (crosses) and EPSR fits to the data (solid line) along with the residuals between data and fit (dotted line) for isotopomers in solution. Roman numerals correspond to sample compositions shown in Table 1, and structure factors are vertically offset by the values listed for clarity.

## Details of the *J*-modulated gHMBC NMR experiment


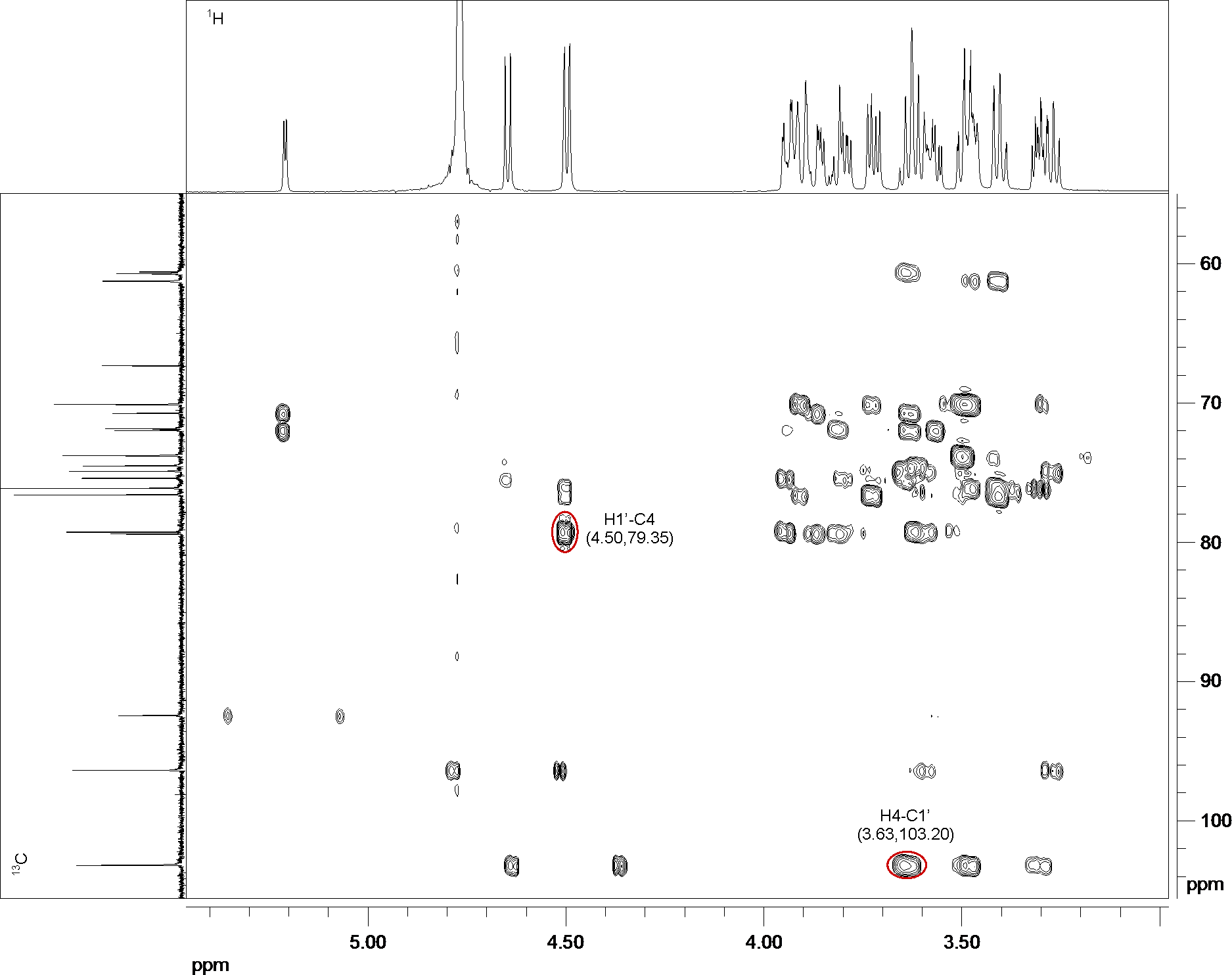


**Figure S2. Example J-modulated gHMBC spectrum acquired from 15 mM cellobiose in D_2_O with a 100 msec *τ* mixing time. Crosspeaks sensitive to (φ,ψ) conformation are highlighted.**

n order to determine the magnitude of the interglycosidic coupling constants ^3^*J*_H1',C4_ and ^3^*J*_H4,C1'_ a J-modulated ^1^H,^13^C gHMBC experiment (J-mod gHMBC) was employed. This experiment, described in detail by Willker and Leibfritz,[5] yields a 2D spectrum showing heteronuclear correlations through two or more bonds with the intensity of each cross-peak depends upon the relationship sin(*π ^n^J_H,X_ τ*) where *τ* represents a variable mixing time for polarization transfer. An example spectrum is shown as Figure S2. Performing a series of experiments with varied times *τ* and integrating the 1-D projection of the cross-peaks of interest from each spectrum produces a set of intensity values as a function of *τ.* J-mod gHMBC Spectra of 15 mM and 0.88 M cellobiose in D_2_O were recorded for values *τ* ranging from 20-200 ms. Resulting data sets were fit to a function of the form of

$y=y_{0}+A\sin(\pi\frac{1}{\omega}(x-x_{c}))$ (1)

where *x* is equivalent to *τ* in ms and ${}^{n}J_{H,X}=\frac{1}{\omega}$ using Origin8.1(OriginLab Corp). Coupling constants were then equated to their relevant torsion angle using the Karplus-type relationship for H–C–O–H dihedrals described by Cloran et al.[6] The cross-peak areas versus *τ* and the non-linear regressions of these data are shown below with the fitting parameters.


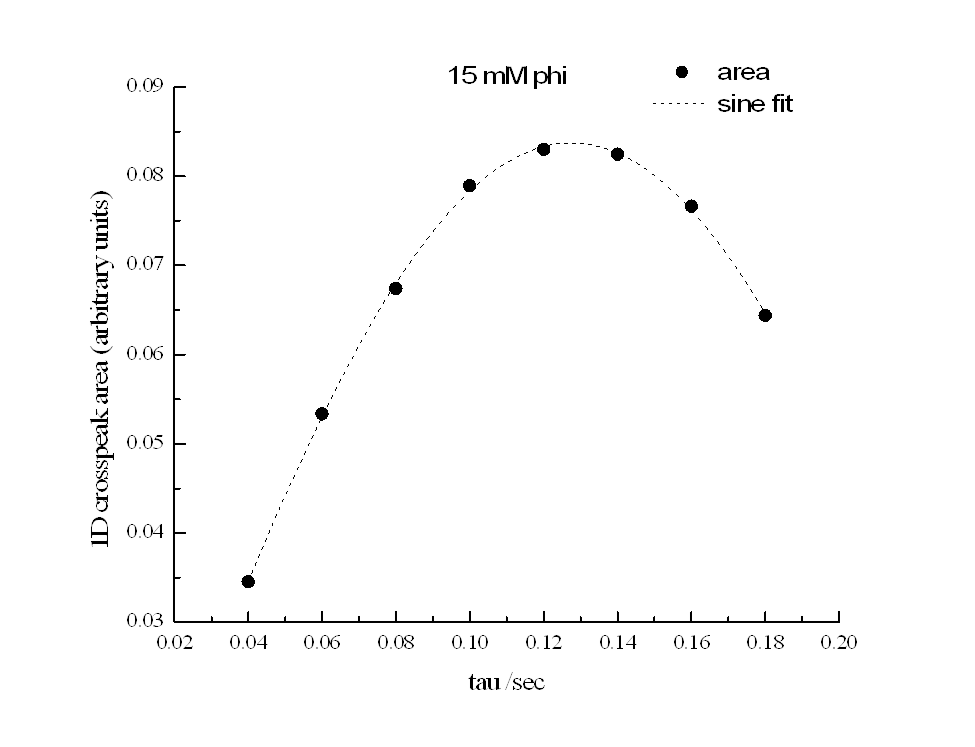


**Figure S3. 15 mM cellobiose J-mod gHMBC crosspeak areas corresponding to ^3^*J*_H1',C4_ as a function of τ.**

**Table S2. Results of sine fit for cross-peak areas corresponding to *^3^J_H1',C4_*** for 15 mM cellobiose.

| Model | Sine |  |  |
| --- | --- | --- | --- |
| Equation | y = y0 + A*sin(pi*(x − xc)/ω) |  |  |
| Reduced Chi-Sqr | 2.86E-07 |  |  |
| Adj. R-Square | 0.99899 |  |  |
|  |  | Value | Standard Error |
| 1D crosspeak area | y0 | 0.0044 | 0.02324 |
| 1D crosspeak area | Xc | 0.01111 | 0.01902 |
| 1D crosspeak area | W | 0.2327 | 0.03784 |
| 1D crosspeak area | A | 0.07933 | 0.02305 |
| 1/ω = 4.30 = ^3^*J*_H1’,C4_ (Hz) | | | |


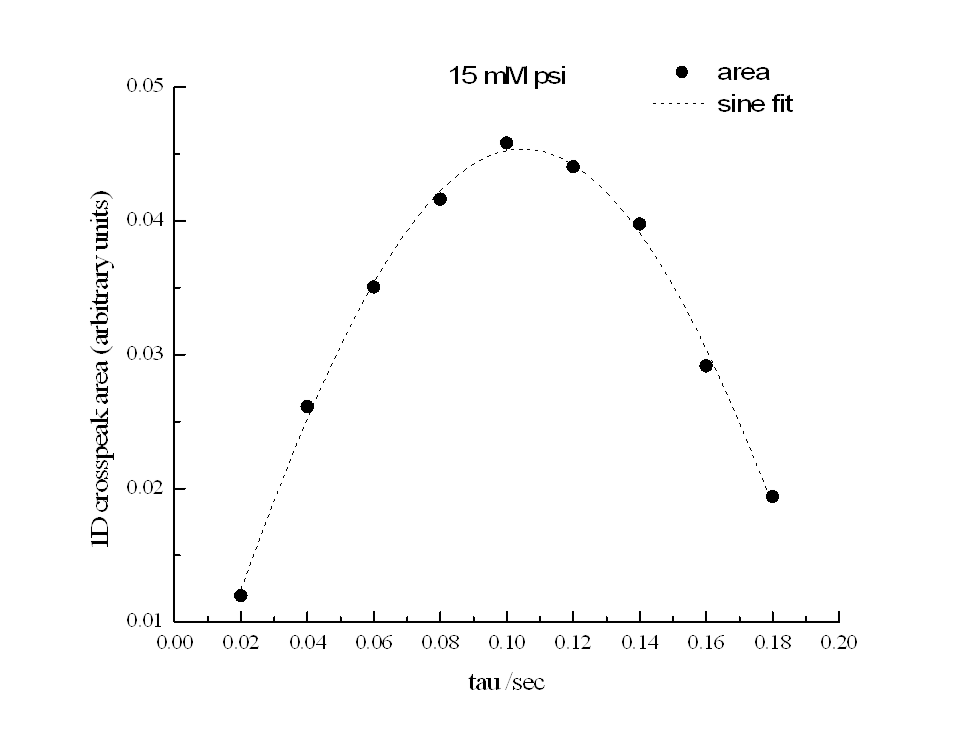


**Figure S4. 15 mM cellobiose J-mod gHMBC crosspeak areas corresponding to ^3^*J*_H4,C1'_ as a function of τ.**

**Table 3. Results of sine fit for crosspeak areas corresponding to *^3^J_H4,C1'_*** for 15 mM cellobiose.

| Model | Sine |  |  |
| --- | --- | --- | --- |
| Equation | y = y0 + A*sin(pi*(x − xc)/ω) |  |  |
| Reduced Chi-Sqr | 8.57E-07 |  |  |
| Adj. R-Square | 0.99376 |  |  |
|  |  | Value | Standard Error |
| 1D crosspeak area | y0 | -0.00764 | 0.0396 |
| 1D crosspeak area | Xc | -0.00788 | 0.04706 |
| 1D crosspeak area | W | 0.2253 | 0.09375 |
| 1D crosspeak area | A | 0.053 | 0.03925 |
| 1/ω = 4.44 *^3^J_H4,C1’_* (Hz) | | | |


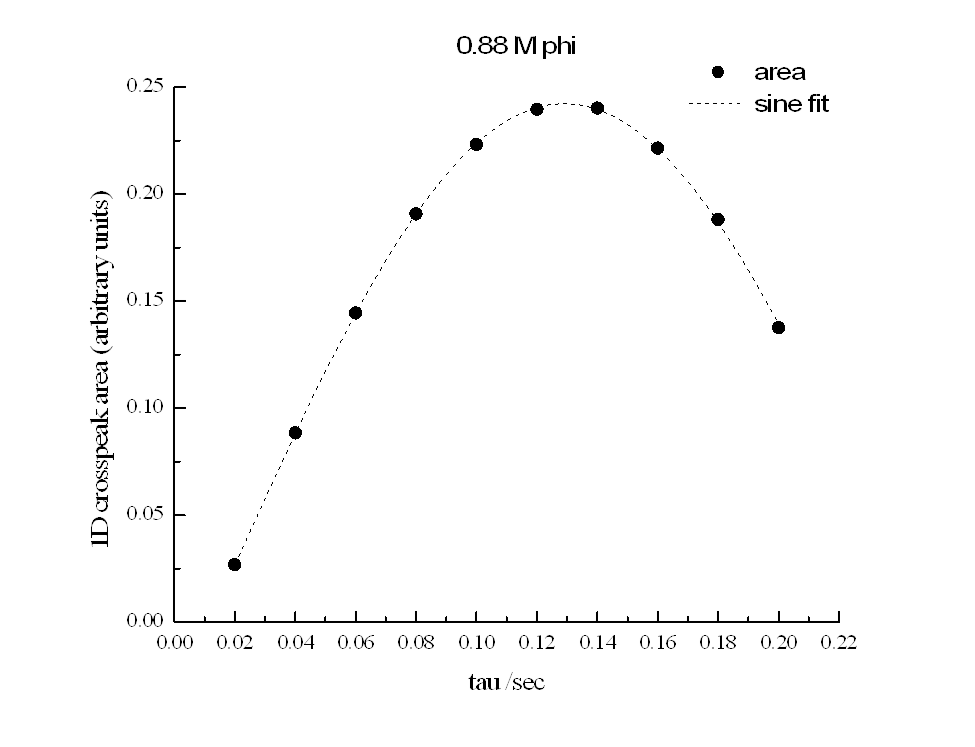


**Figure S5. 0.88 M Cellobiose J-mod gHMBC crosspeak areas corresponding to ^3^*J*_H1',C4 '_as a function of τ.**

**Table S4. Results of sine fit for cross-peak areas corresponding to *^3^J_H1',C4_*** for 0.88 M cellobiose.

| Model | Sine |  |  |
| --- | --- | --- | --- |
| Equation | y = y0 + A*sin(pi*(x − xc)/ω) |  |  |
| Reduced Chi-Sqr | 9.77E-07 |  |  |
| Adj. R-Square | 0.9998 |  |  |
|  |  | Value | Standard Error |
| 1D crosspeak area | y0 | 0.02642 | 0.01212 |
| 1D crosspeak area | Xc | 0.01987 | 0.0037 |
| 1D crosspeak area | W | 0.21826 | 0.00731 |
| 1D crosspeak area | A | 0.2158 | 0.01183 |
| 1/ω = 4.58 = *^3^J_H1’,C4_* (Hz) | | | |


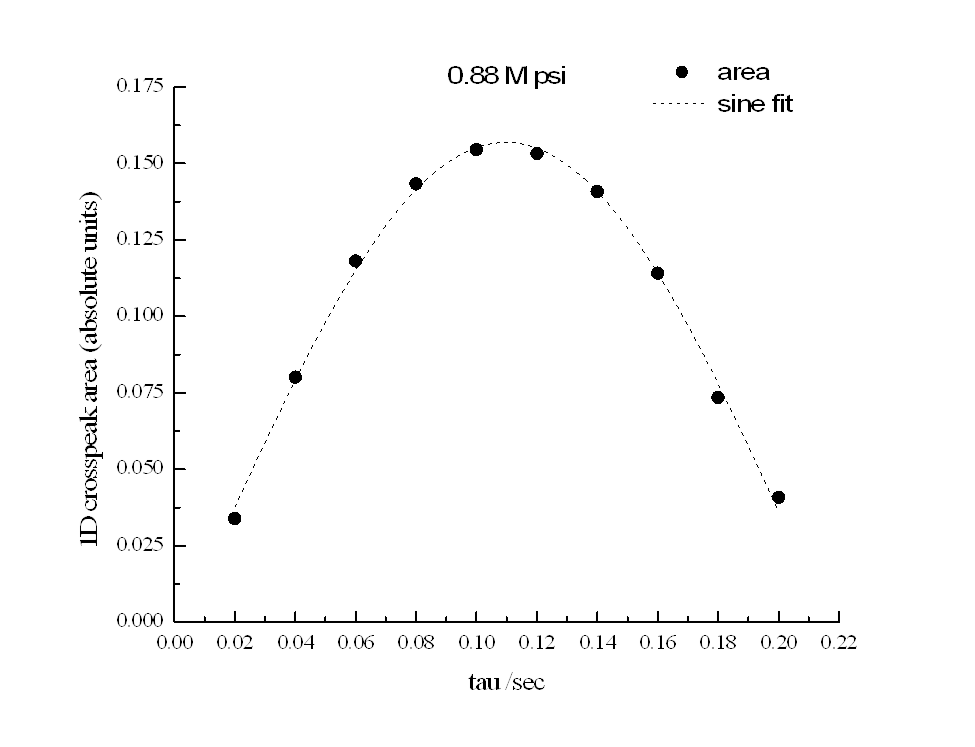


**Figure S6. 0.88 M Cellobiose J-mod gHMBC crosspeak areas corresponding to *^3^J_H4,C1'_*as a function of τ.**

**Table S5. Results of sine fit for crosspeak areas corresponding to *^3^J_H4,C1'_*** for 0.88 M cellobiose.

| Model | Sine |  |  |
| --- | --- | --- | --- |
| Equation | y = y0 + A*sin(pi*(x − xc)/ω) |  |  |
| Reduced Chi-Sqr | 1.17E-05 |  |  |
| Adj. R-Square | 0.99432 |  |  |
|  |  | Value | Standard Error |
| 1D crosspeak area | y0 | 0.02852 | 0.04381 |
| 1D crosspeak area | Xc | 0.01578 | 0.01935 |
| 1D crosspeak area | W | 0.18781 | 0.03869 |
| 1D crosspeak area | A | 0.12841 | 0.04261 |
| 1/ω = 5.32 = *^3^J_H4,C1’_* | | | |

## EPSR model parameters

Lennard-Jones parameters (σ in Å and ε in kJ mol^-1^) and partial charges for defining non-bonding interaction potentials were taken from the literature; specifically the SPC/E model[7] was used for the water atoms and the modified CHARMM force field for carbohydrates developed by Guvench et al. was used for the atoms of the cellobiose molecules (Table S6).[8]

**Table S6. Lennard-Jones parameters and charges used to define the reference potential for EPSR simulation.**

| atom type | ε(kJ/mol) | σ(Å) | charge(e) |
| --- | --- | --- | --- |
| O1 | 0.14840 | 2.93997 | -0.40000 |
| O1p | 0.14840 | 2.93997 | -0.40000 |
| OL | 0.14840 | 2.93997 | -0.40000 |
| C | 0.13389 | 3.56359 | 0.14000 |
| O | 0.80375 | 3.14487 | -0.65000 |
| Op | 0.80375 | 3.14487 | -0.65000 |
| Oi | 0.80375 | 3.14487 | -0.65000 |
| H | 0 | 0 | 0.42380 |
| Hp | 0 | 0 | 0.42380 |
| Hi | 0 | 0 | 0.42380 |
| M | 0.18828 | 2.38761 | 0.09714 |
| Ow |  |  |  |
| Hw | 0 | 0 | 0.42380 |

## EPSR torsion angle constraints

The ^4^*C*_1_ hexopyranose conformation was imposed in the EPSR model for both α- and β-cellobiose by specifying all backbone carbon torsion angles to match those of the β-cellobiose crystal structure.[9] Table S7 details the angles and their values. It should be noted that these torsions do not include any of the form X–X–O–H that could potentially influence hydroxyl–water hydrogen bonding. Furthermore, given the availability of data describing the conformation of the glycosidic linkage, a second set of torsional constraints was added to the EPSR model so that the values of φ_H_ and ψ_H_ match those derived from the J-mod gHMBC experiments performed with 1:63 cellbiose:D_2_O. The EPSR routine converts all specified torsion angles to non-bonded distance constraints between atoms one and four of the torsions; this approach allowed for flexibility of at least ± 30° in each torsion.

**Table S7. Torsion angles specific to ^4^*C*_1_** chair conformation specified for cellobiose molecules in the EPSR simulation.

| α-cellobiose | | | | β-cellobiose | | | |
| --- | --- | --- | --- | --- | --- | --- | --- |
| non-reducing ring | | reducing ring | | non-reducing ring | | reducing ring | |
| atoms | angle | atoms | angle | atoms | angle | atoms | angle |
| C1'-C2'-C3'-C4' | -057.27 | C1-C2-C3-C4 | -050.66 | C1'-C2'-C3'-C4' | -057.27 | C1-C2-C3-C4 | -050.66 |
| C1'-C2'-C3'-H3' | 059.09 | C1-C2-C3-H3 | 070.02 | C1'-C2'-C3'-H3' | 059.09 | C1-C2-C3-H3 | 070.02 |
| C1'-C2'-C3'-O3' | 178.53 | C1-C2-C3-O3 | -174.35 | C1'-C2'-C3'-O3' | 178.53 | C1-C2-C3-O3 | -174.35 |
| C2'-C1'-O5'-C6' | -065.71 | C2-C1-O5-C6 | -065.05 | C2'-C1'-O5'-C6' | -065.71 | C2-C1-O5-C6 | -065.05 |
| C2'-C3'-C4'-C5' | 051.99 | C2-C3-C4-C5 | 047.98 | C2'-C3'-C4'-C5' | 051.99 | C2-C3-C4-C5 | 047.98 |
| C2'-C3'-C4'-H4' | -071.87 | C2-C3-C4-H4 | -072.57 | C2'-C3'-C4'-H4' | -071.87 | C2-C3-C4-H4 | -072.57 |
| C2'-C3'-C4'-O4' | 171.94 |  |  | C2'-C3'-C4'-O4' | 171.94 |  |  |
| C3'-C4'-C5'-C6' | -168.51 | C3-C4-C5-C6 | -169.68 | C3'-C4'-C5'-C6' | -168.51 | C3-C4-C5-C6 | -169.68 |
| C3'-C4'-C5'-H5' | 069.41 | C3-C4-C5-H5 | 068.85 | C3'-C4'-C5'-H5' | 069.41 | C3-C4-C5-H5 | 068.85 |
| C3'-C4'-C5'-O5' | -051.95 | C3-C4-C5-O5 | -051.14 | C3'-C4'-C5'-O5' | -051.95 | C3-C4-C5-O5 | -051.14 |
| C4'-C5'-O5'-C1' | 059.80 | C4-C5-O5-C1 | 060.90 | C4'-C5'-O5'-C1' | 059.80 | C4-C5-O5-C1 | 060.90 |
| C6'-C5'-O5'-C1' | 179.75 | C6-C5-O5-C1 | -176.15 | C6'-C5'-O5'-C1' | 179.75 | C6-C5-O5-C1 | -176.15 |
| H1'-C1'-C2'-C3' | -055.38 | H1-C1-C2-C3 | 175.07 | H1'-C1'-C2'-C3' | -055.38 | H1-C1-C2-C3 | -073.66 |
| H1'-C1'-C2'-H2' | -177.93 | H1-C1-C2-H2 | 054.85 | H1'-C1'-C2'-H2' | -177.93 | H1-C1-C2-H2 | 166.12 |
| H1'-C1'-C2'-O2 | 069.41 | H1-C1-C2-O2 | -067.64 | H1'-C1'-C2'-O2 | 069.41 | H1-C1-C2-O2 | 043.62 |
| H1'-C1'-O5'-C6' | 055.49 | H1-C1-O5-C6 | 175.61 | H1'-C1'-O5'-C6' | 055.49 | H1-C1-O5-C6 | 060.08 |
| H2'-C2'-C3'-C4' | 064.06 | H2-C2-C3-C4 | 069.80 | H2'-C2'-C3'-C4' | 064.06 | H2-C2-C3-C4 | 069.80 |
| H2'-C2'-C3'-H3' | -179.57 | H2-C2-C3-H3 | -169.52 | H2'-C2'-C3'-H3' | -179.57 | H2-C2-C3-H3 | -169.52 |
| H2'-C2'-C3'-O3' | -060.14 | H2-C2-C3-O3 | -053.89 | H2'-C2'-C3'-O3' | -060.14 | H2-C2-C3-O3 | -053.89 |
| H3'-C3'-C4'-C5' | -057.63 | H3-C3-C4-C5 | -076.44 | H3'-C3'-C4'-C5' | -057.63 | H3-C3-C4-C5 | -076.44 |
| H3'-C3'-C4'-H4' | 178.51 | H3-C3-C4-H4 | 163.01 | H3'-C3'-C4'-H4' | 178.51 | H3-C3-C4-H4 | 163.01 |
| H3'-C3'-C4'-O4' | 062.32 |  |  | H3'-C3'-C4'-O4' | 062.32 |  |  |
| H4'-C4'-C5'-C6' | -049.20 | H4-C4-C5-C6 | -045.22 | H4'-C4'-C5'-C6' | -049.20 | H4-C4-C5-C6 | -045.22 |
| H4'-C4'-C5'-H5' | -171.28 | H4-C4-C5-H5 | -166.69 | H4'-C4'-C5'-H5' | -171.28 | H4-C4-C5-H5 | -166.69 |
| H4'-C4'-C5'-O5' | 067.36 | H4-C4-C5-O5 | 073.32 | H4'-C4'-C5'-O5' | 067.36 | H4-C4-C5-O5 | 073.32 |
| H5'-C5'-O5'-C1' | -068.27 | H5-C5-O5-C1 | -053.33 | H5'-C5'-O5'-C1' | -068.27 | H5-C5-O5-C1 | -053.33 |
|  |  | O1-C1-C2-C3 | -073.66 |  |  | O1-C1-C2-C3 | 175.07 |
|  |  | O1-C1-C2-H2 | 166.12 |  |  | O1-C1-C2-H2 | 054.85 |
|  |  | O1-C1-C2-O2 | 043.62 |  |  | O1-C1-C2-O2 | -067.64 |
|  |  | O1-C1-O5-C6 | 060.08 |  |  | O1-C1-O5-C6 | 175.61 |
| O2'-C2'-C3'-C4' | -179.93 | O2-C2-C3-C4 | -170.52 | O2'-C2'-C3'-C4' | -179.93 | O2-C2-C3-C4 | -170.52 |
| O2'-C2'-C3'-H3' | -063.57 | O2-C2-C3-H3 | -049.84 | O2'-C2'-C3'-H3' | -063.57 | O2-C2-C3-H3 | -049.84 |
| O2'-C2'-C3'-O3' | 055.87 | O2-C2-C3-O3 | 065.80 | O2'-C2'-C3'-O3' | 055.87 | O2-C2-C3-O3 | 065.80 |
| O3'-C3'-C4'-C5' | 176.51 | O3-C3-C4-C5 | 168.61 | O3'-C3'-C4'-C5' | 176.51 | O3-C3-C4-C5 | 168.61 |
| O3'-C3'-C4'-H4' | 052.66 | O3-C3-C4-H4 | 048.07 | O3'-C3'-C4'-H4' | 052.66 | O3-C3-C4-H4 | 048.07 |
| O3'-C3'-C4'-O4' | -063.54 |  |  | O3'-C3'-C4'-O4' | -063.54 |  |  |
| O4'-C4'-C5'-C6' | 072.29 |  |  | O4'-C4'-C5'-C6' | 072.29 |  |  |
| O4'-C4'-C5'-H5' | -049.79 |  |  | O4'-C4'-C5'-H5' | -049.79 |  |  |
| O4'-C4'-C5'-O5' | -171.15 |  |  | O4'-C4'-C5'-O5' | -171.15 |  |  |
| O5'-C1'-C2'-C3' | 063.54 | O5-C1-C2-C3 | 057.80 | O5'-C1'-C2'-C3' | 063.54 | O5-C1-C2-C3 | 057.80 |
| O5'-C1'-C2'-H2' | -059.01 | O5-C1-C2-H2 | -062.42 | O5'-C1'-C2'-H2' | -059.01 | O5-C1-C2-H2 | -062.42 |
| O5'-C1'-C2'-O2' | -171.66 | O5-C1-C2-O2 | 175.09 | O5'-C1'-C2'-O2' | -171.66 | O5-C1-C2-O2 | 175.09 |

In an ideal ^4^*C*_1_ chair the torsions O5–C1–C2–C3, C2–C3–C4–C5 and C4–C5–O5–C1 assume values of ~55°. Monitoring the sum of these torsions for each ring (Figure S7) revealed average values ranging from 51°-57° as determined from fitting the values to normal distributions (Table S8). The Range of values observed shows that none of the cellobiose molecules deviated from a chair-like conformation throughout the course of EPSR simulation.


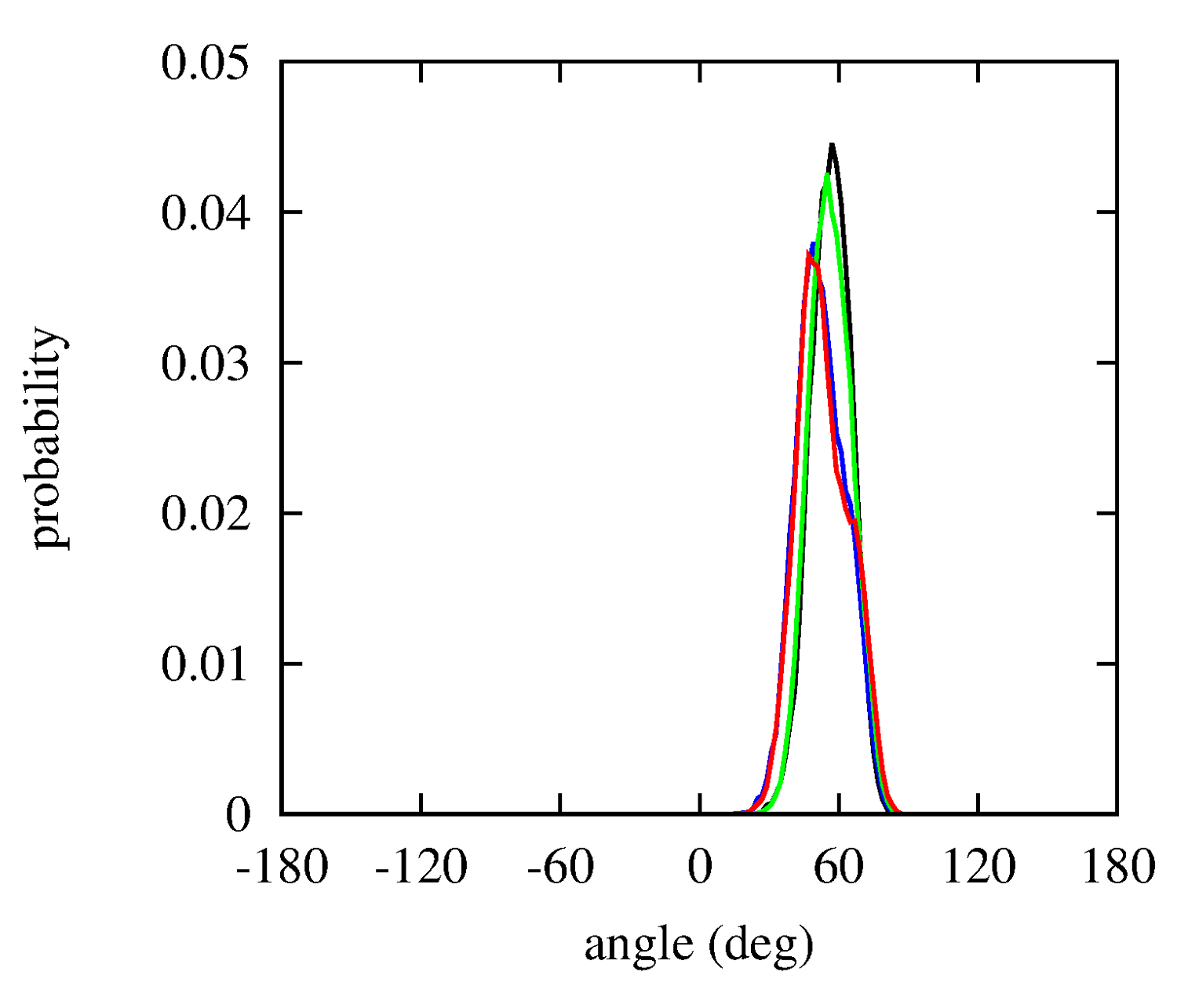


**Figure S7. Summed positive chair-like torsions for glucopyranose rings in cellobiose.** Values for the reducing and non-reducing rings of α-cellobiose are shown in blue and black respectively; values for the reducing and non-reducing rings of β-cellobiose are shown in red and green, respectively.

**Table S8.** Normal distribution approximations of summed positive chair-like torsions of cellobiose.

| Anomer | Ring | Average chair torsion (°) | | s.d. (°) |
| --- | --- | --- | --- | --- |
| α-cellobiose | reducing | 51.96 | 11.21 | |
|  | non-reducing | 56.86 | 8.94 | |
| β-cellobiose | Reducing | 52.29 | 11.82 | |
|  | non-reducing | 56.17 | 9.60 | |

Furthermore, the torsion angles specific to the glycosidic linkage in cellobiose were also specified to match those determined from the J-modulated gHMBC experiments described above. Since the EPSR routine converts specified torsion angles to non-bonding distance constraints, it was necessary to constrain all possible torsions across the linkage. The torsions which were defined are shown in Table S9.

**Table S9.** Torsion angles specific to the cellobiose glycosidic linkage conformation specified in the EPSR simulation.

| Defining atoms | Dihedral angle (°) |
| --- | --- |
| H1'-C1'-O4-C4 | 33.30 |
| C2'-C1'-O4-C4 | 157.52 |
| O5'-C1'-O4-C4 | -85.32 |
| C1'-O4-C4'-H4' | -39.70 |
| C1'-O4-C4-C3 | 084.56 |
| C1'-O4-C4-C5 | -154.12 |

## Additional RDFs from EPSR fits to the neutron diffraction data.

As stated in the main text, the primary advantage of the EPSR method for interpreting NDIS data is the ability to extract all radial distribution functions from the model. Intermolecular radial distribution functions derived from the EPSR model and not shown in the main text are shown below in Figures S8-S19.


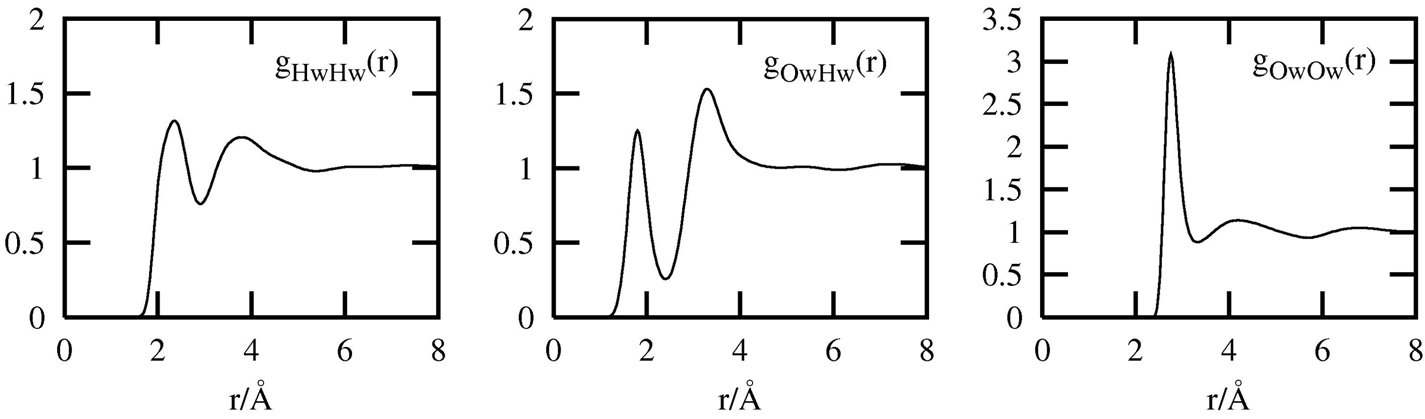


**Figure S8. Radial distribution functions corresponding to bulk water structure.**


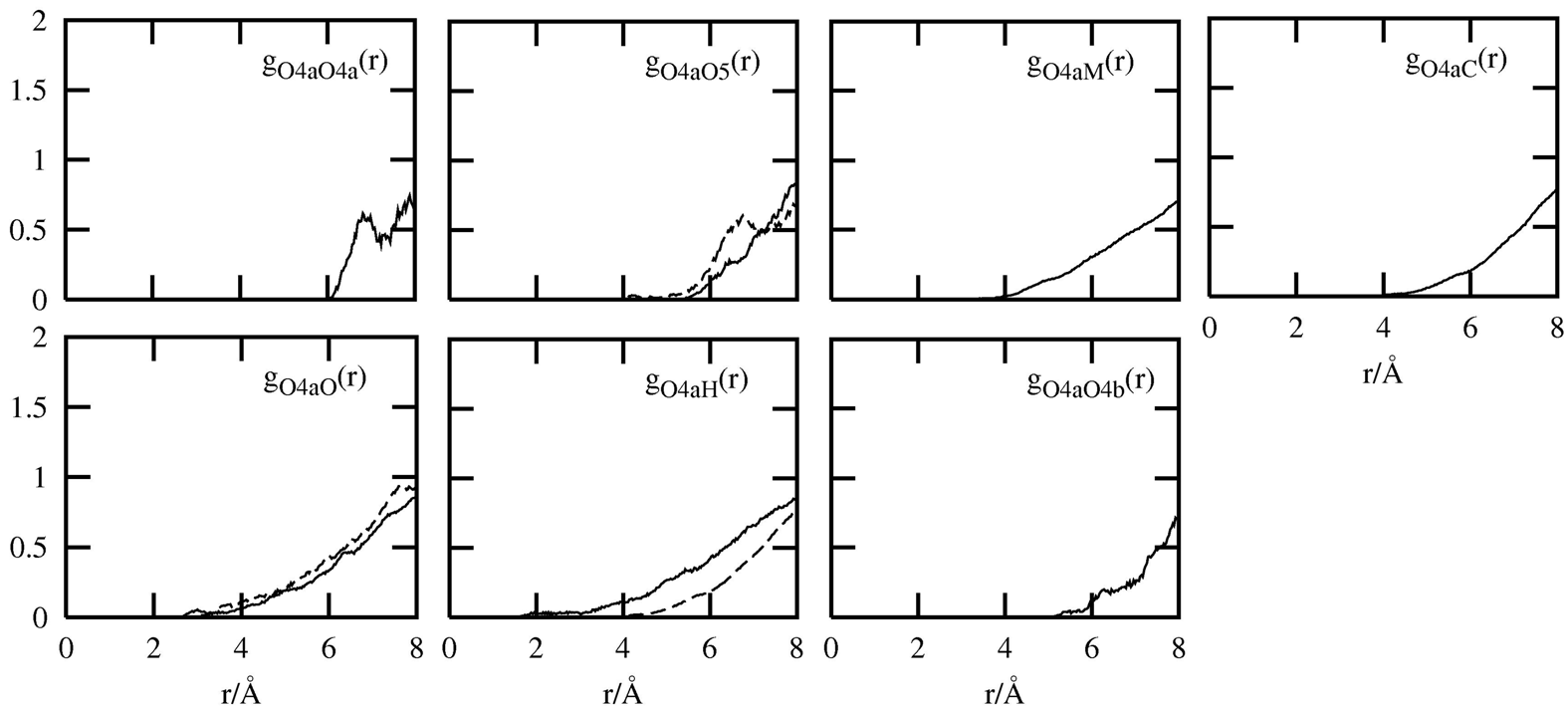


**Figure S9. Radial distribution functions for additional O4a–X atom pairs.**

**
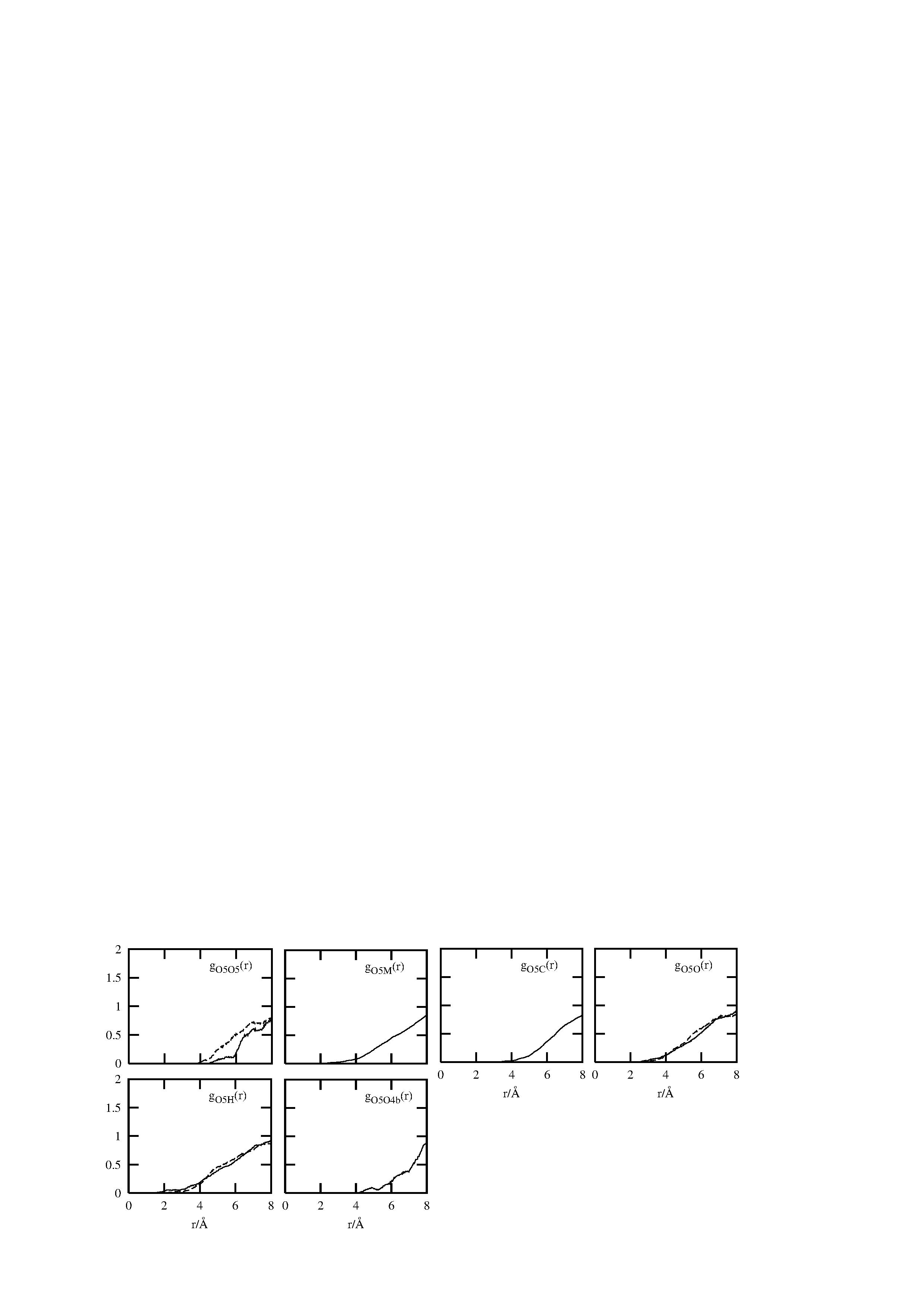
**

**Figure S10. Radial distribution functions for additional O5–X atom pairs.**

**
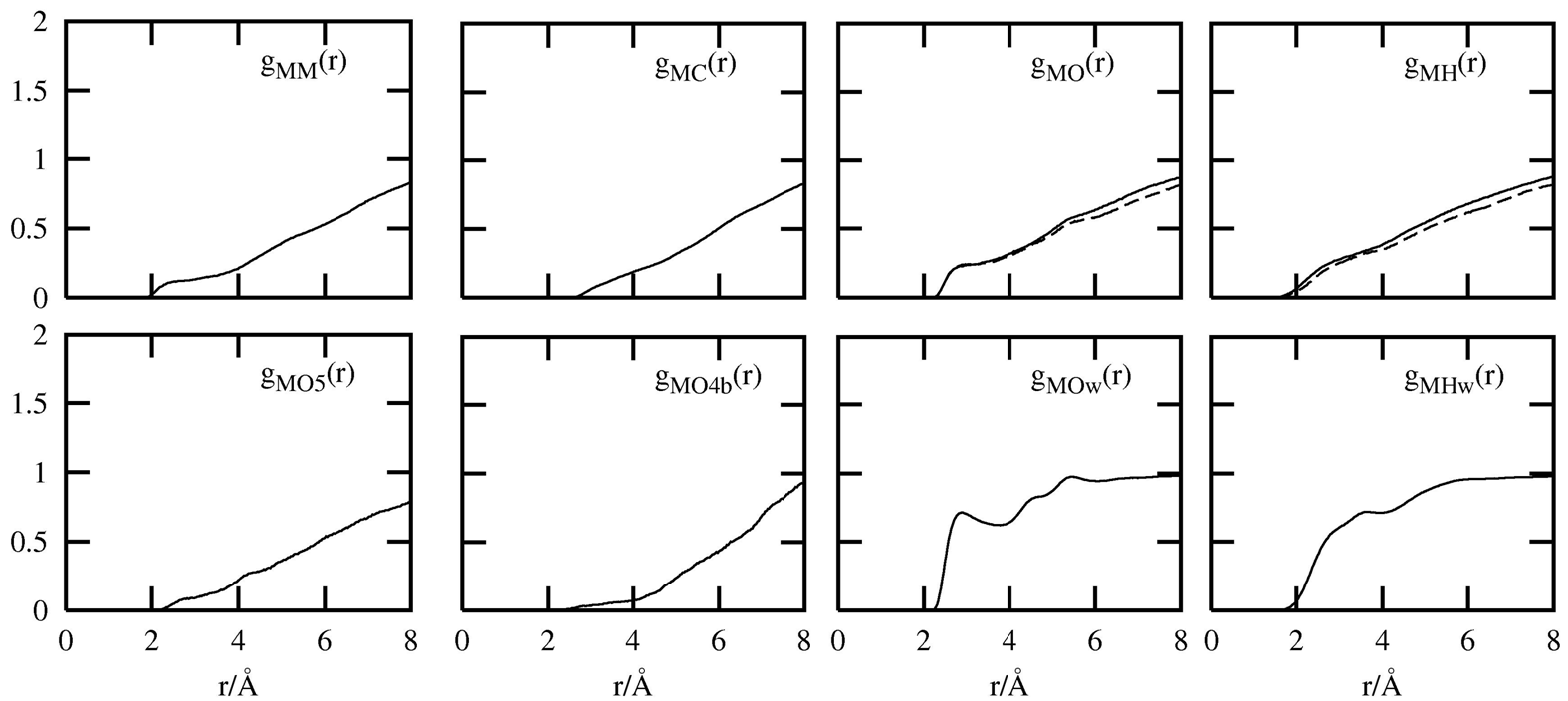
**

**Figure S11. Radial distribution functions for additional M–X atom pairs.**

**
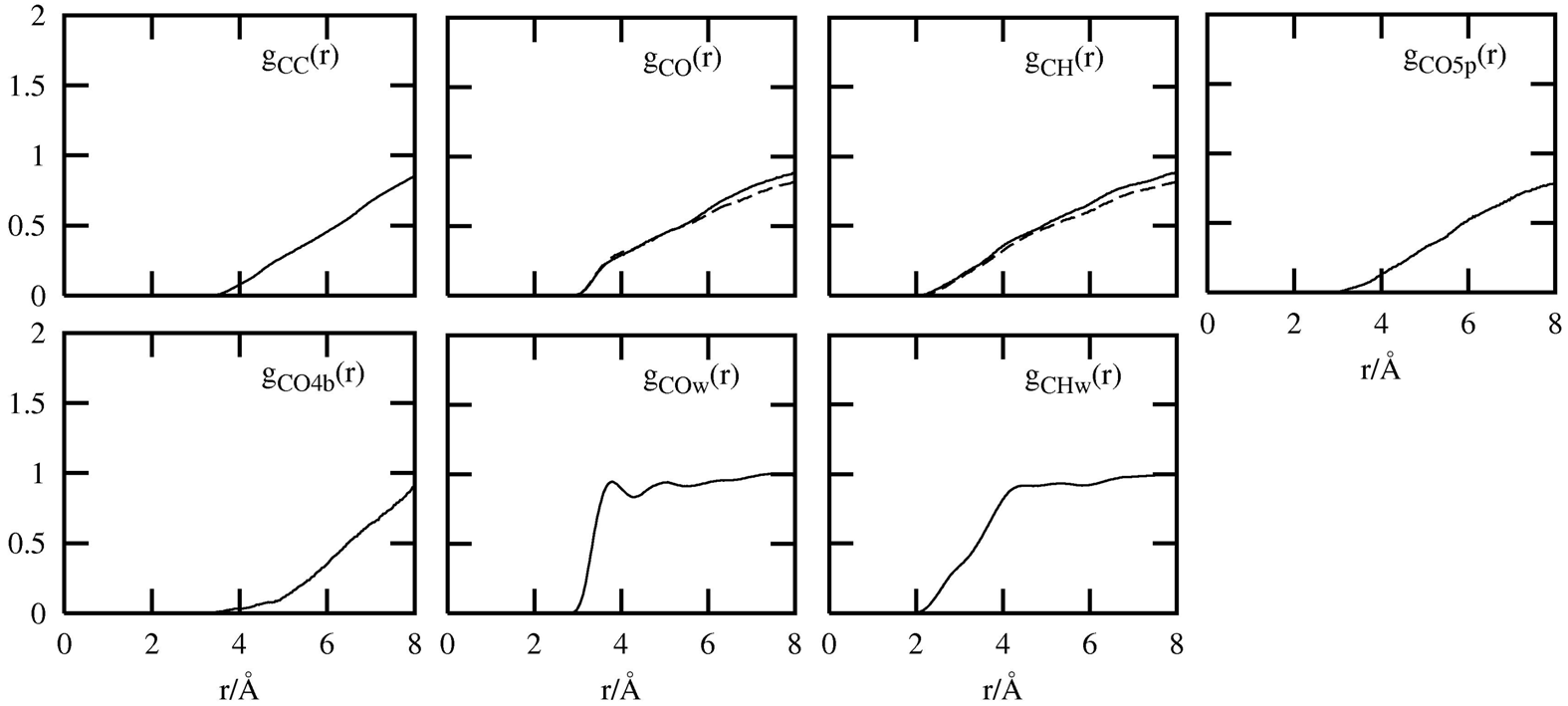
**

**Figure S12. Radial distribution functions for additional C–X atom pairs.**


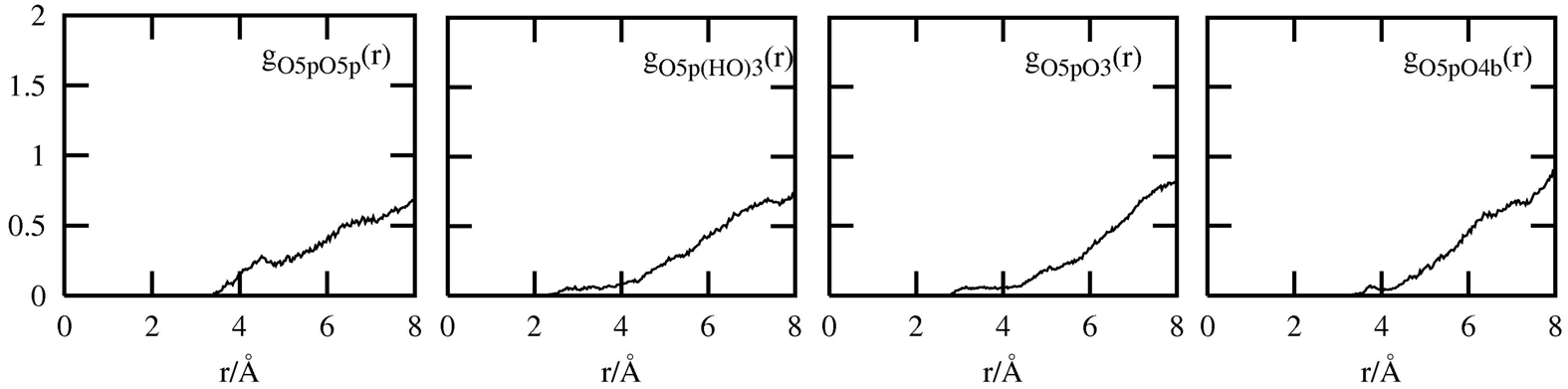


**Figure S13. Radial distribution functions for additional O5p–X atom pairs.**

**
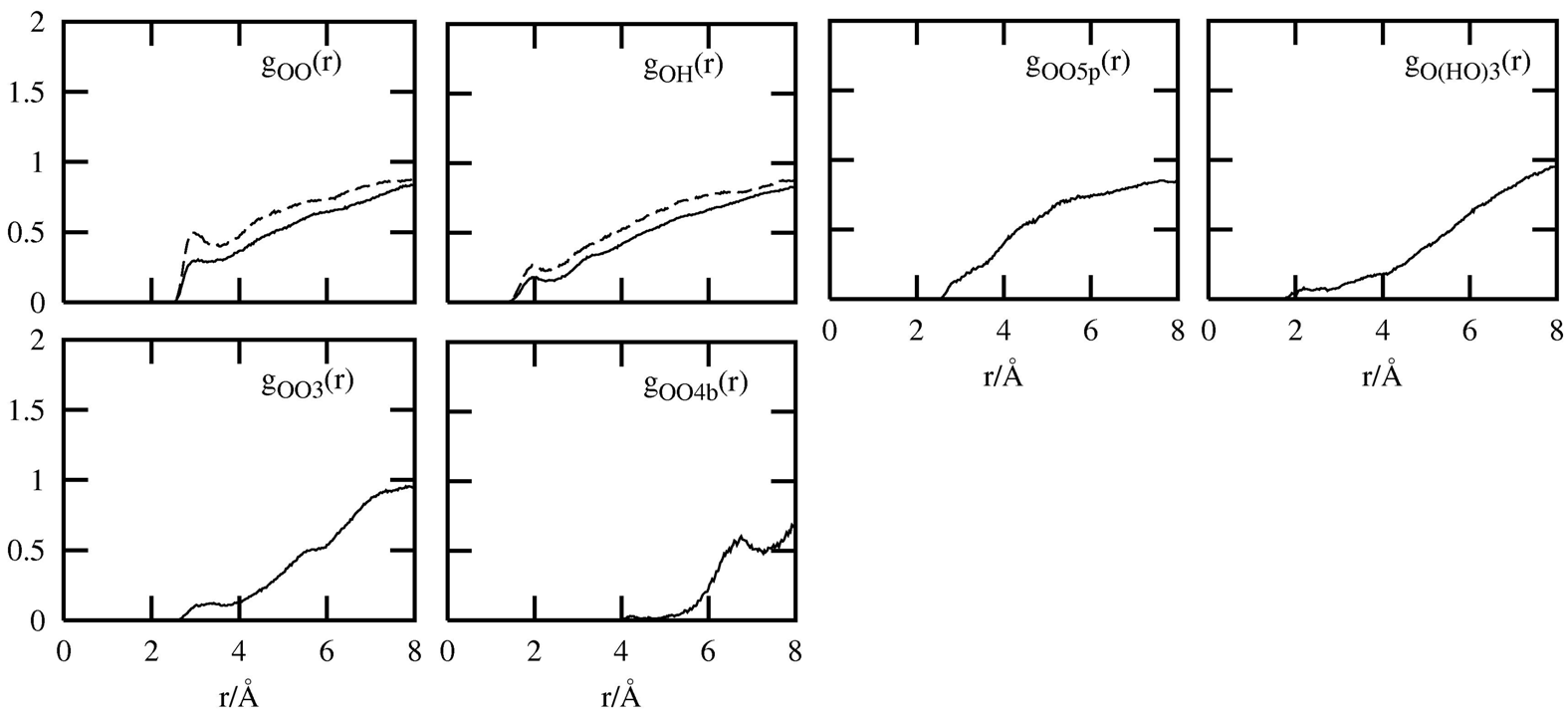
**

**Figure S14. Radial distribution functions for additional O–X atom pairs.**

**
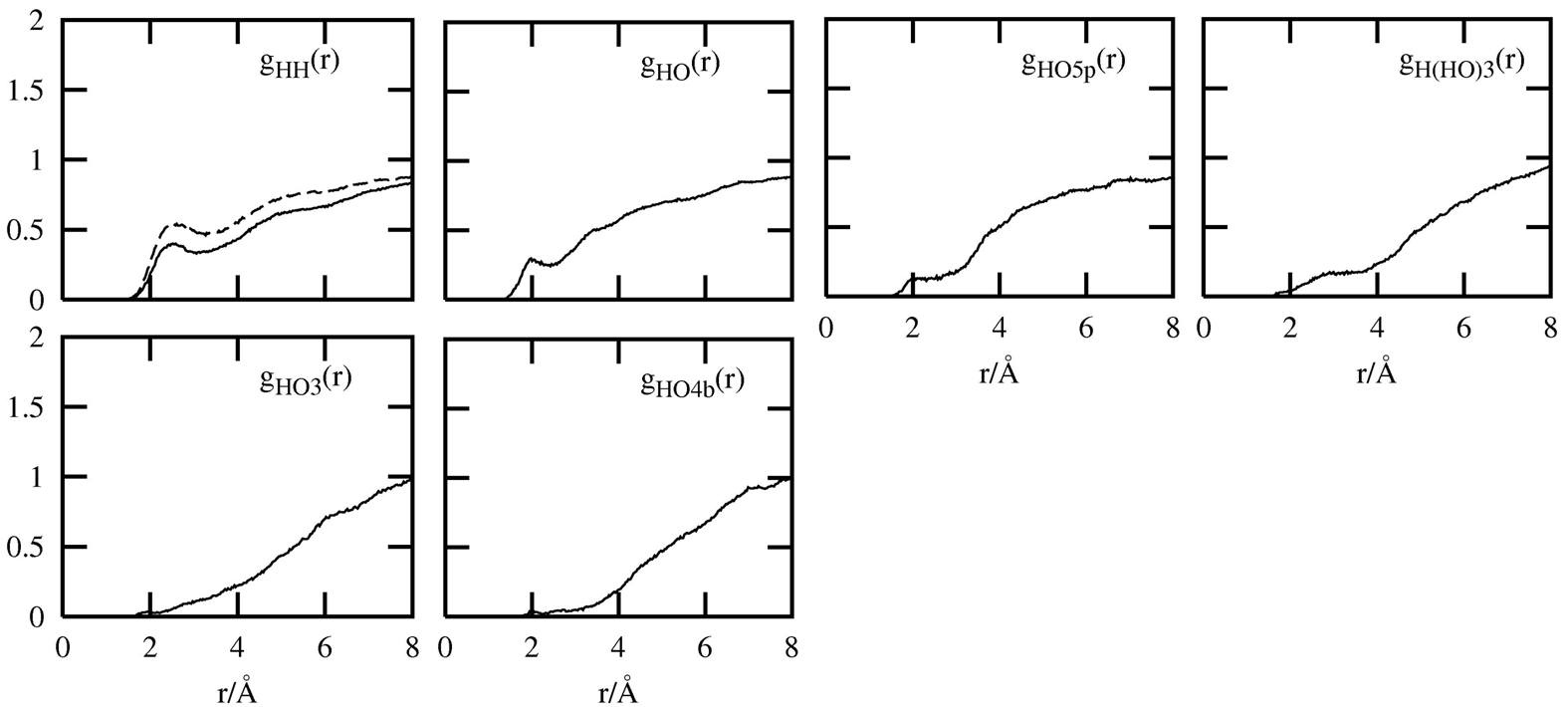
**

**Figure S15. Radial distribution functions for additional H–X atom pairs.**

**
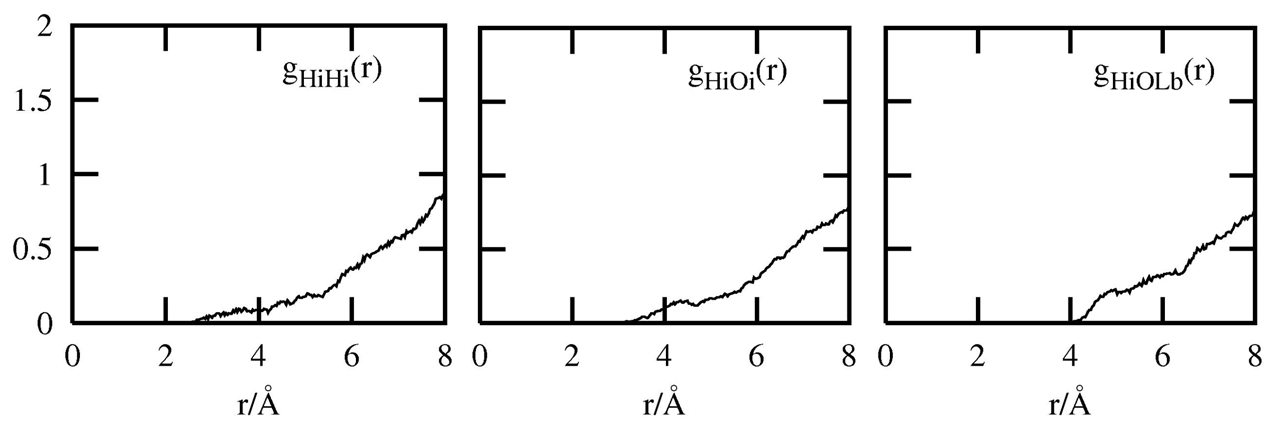
**

**Figure S16. Radial distribution functions for additional Hi–X atom pairs.**

**
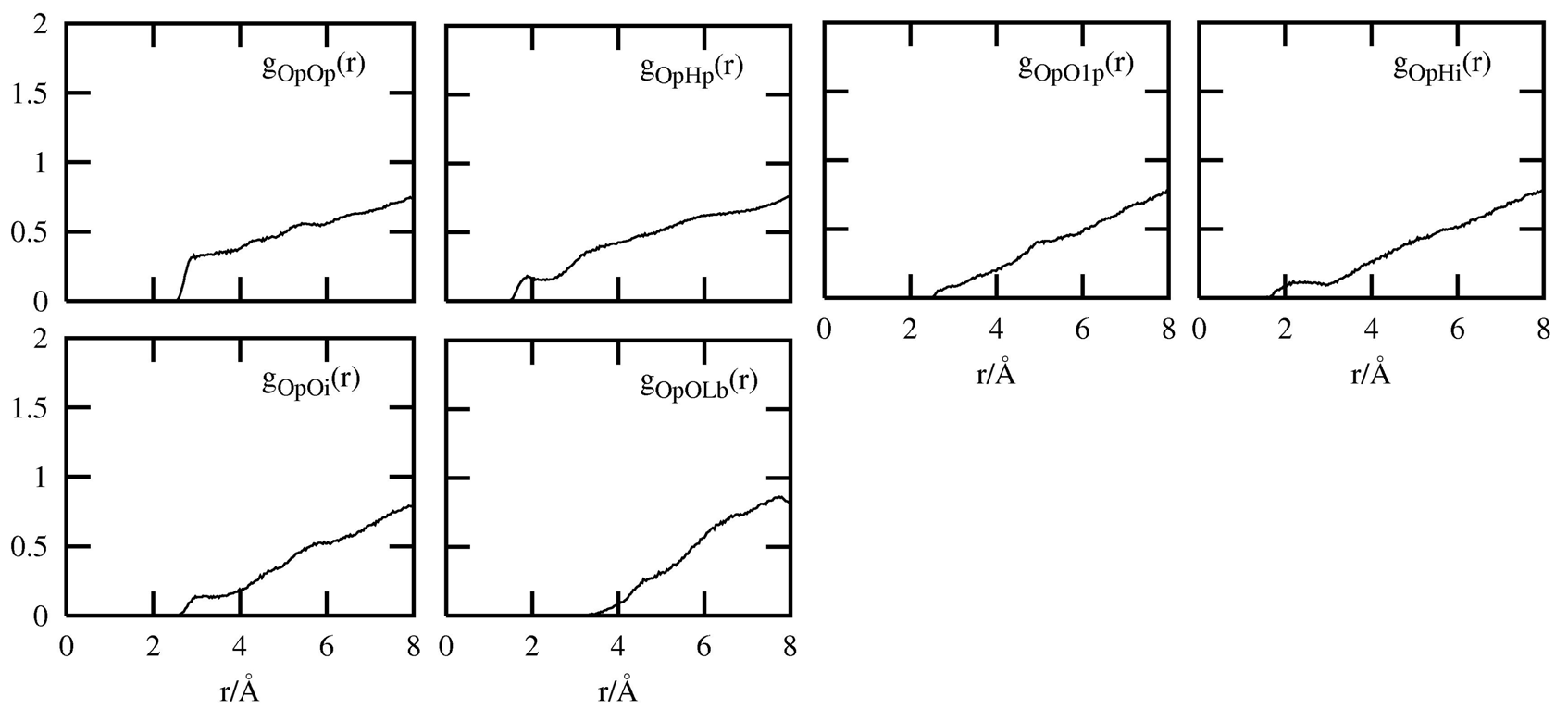
**

**Figure S17. Radial distribution functions for additional Op–X atom pairs.**

**
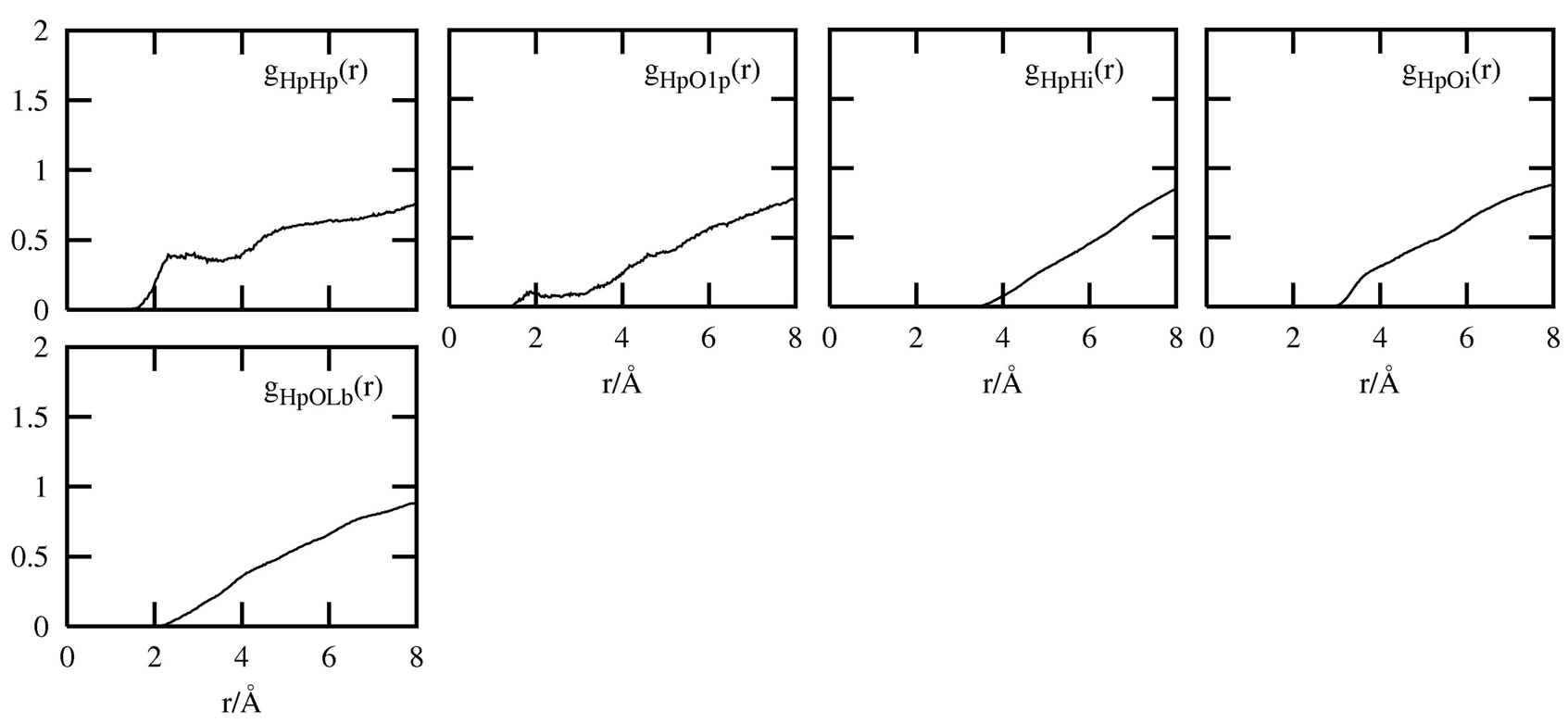
**

**Figure S18. Radial distribution functions for additional Hp–X atom pairs.**

**
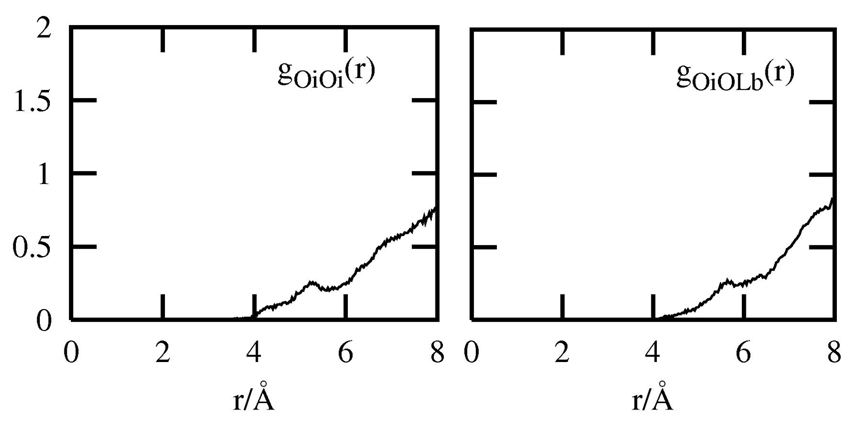
** **
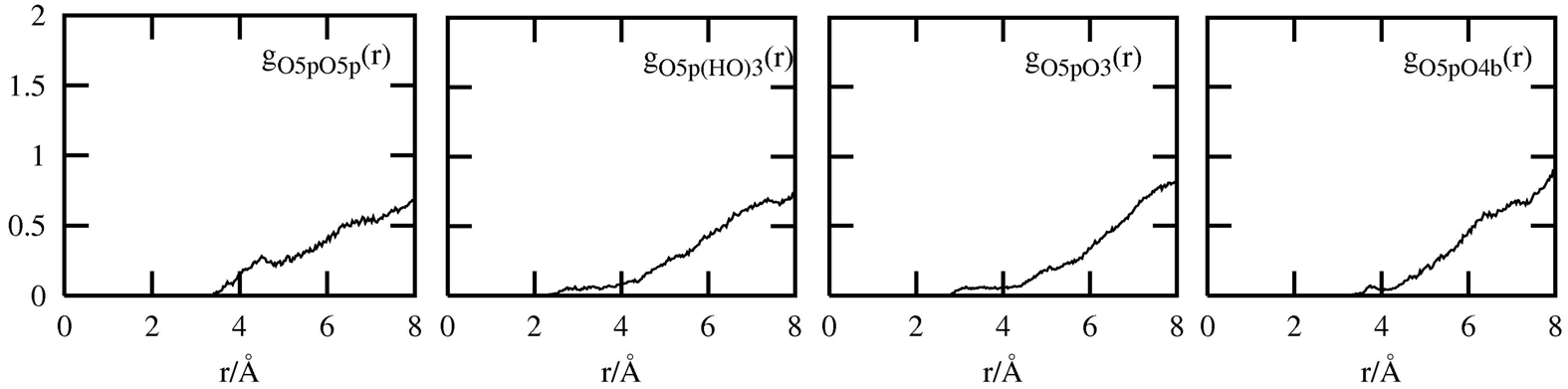
**

**Figure S19. Radial Distribution functions for additional Oi–X and O5b–X atom pairs.**

## References

1. Svishchev IM, Kusalik PG (1993) Structure in Liquid Water - a Study of Spatial-Distribution Functions. J Chem Phys 99: 3049-3058.

2. Svishchev IM, Kusalik PG (1993) Roto-Translational Motion in Liquid Water and Its Structural Implication. Chem Phys Lett 215: 596-600.

3. Soper AK (1994) Orientational correlation function for molecular liquids: The case of liquid water. J Chem Phys 101: 6888-6901.

4. Gray CG, Gubbins KE (1984) Theory of Molecular Liquids Vol I: Fundamentals. New York: Oxford University Press.

5. Willker W, Leibritz D (1995) Determination of Heteronuclear Long-Range H,X Coupling Constants from Gradient-Selected HMBC Spectra. Magn Reson Chem 33: 632-638.

6. Cloran F, Carmichael I, Serianni AS (1999) Density Functional Calculations on Disaccharide Mimics: Studies of Molecular Geometries and Trans-*O*-glycosidic *^3^J_COCH_* and *^3^J_COCC_* Spin-Couplings. J Am Chem Soc 121: 9843-9851.

7. Berendsen HJC, Grigera JR, Straatsma TP (1987) The missing term in effective pair potentials. J Phys Chem 91: 6269-6271.

8. Guvench O, Greene SN, Kamath G, Brady JW, Venable RM, et al. (2008) Additive Empirical Force Field for Hexopyranose Monosaccharides. J Comput Chem 29: 2543-2564.

9. Chu SSC, Jeffrey GA (1968) Refinement of crystal structures of β-D-glucose and cellobiose. Acta Crystallogr, Sect B: Struct Sci 24: 830-838.
